# From Aquifer to Tap: Comprehensive Quali-Quantitative Evaluation of Plastic Particles Along a Drinking Water Supply Chain of Milan (Northern Italy)

**DOI:** 10.3390/jox16010018

**Published:** 2026-01-22

**Authors:** Andrea Binelli, Alberto Cappelletti, Cristina Cremonesi, Camilla Della Torre, Giada Caorsi, Stefano Magni

**Affiliations:** Department of Biosciences, University of Milan, Via Celoria 26, 20133 Milan, Italy or alberto.cappelletti.t@gmail.com (A.C.); cristina.cremonesi@unimi.it (C.C.); camilla.dellatorre@unimi.it (C.D.T.); giada.caorsi@unimi.it (G.C.)

**Keywords:** ecology, polymers, monitoring, drinking water, spectroscopy

## Abstract

This study presents the first evaluation of plastic particle contamination along a complete drinking water supply chain within the distribution system of Milan, Northern Italy. Fourteen grab water samples were collected from various points, including groundwater extraction, post-treatment stages, a public fountain, and ten household taps. Plastic particles were identified using µFTIR spectroscopy and characterized by polymer type, shape, size, and color. Overall, low concentrations of plastic particles were detected, ranging from 0.3 ± 0.5 particles/L in the accumulation tank to an average of 1.9 ± 1.4 particles/L in household tap water, with no significant increase observed along the supply chain. The entire data set was dominated by cellulose particles (76%), as plastics accounted for only 8%. Microplastics (1 µm–1 mm) were the most commonly detected (90%), while the remaining 10% were large microplastics (1–5 mm). Qualitatively, polyester fibers were the most prevalent particles identified. However, greater variability and higher concentrations were found in private residence samples, suggesting that internal plumbing systems may be a primary source of contamination. Estimated human exposure through this supply system, based on a current theoretical model, was minimal compared to global benchmarks. These findings highlight the necessity of integrating private distribution infrastructure into future regulatory frameworks to assist stakeholders in making informed decisions to mitigate potential plastic contamination.

## 1. Introduction

Water is an essential resource for human life, serving not only as a source of drinking water but also playing crucial roles in domestic (e.g., cooking, hygiene, recreation), industrial (e.g., transportation, energy production, manufacturing), and agricultural (e.g., irrigation, pesticide application) activities [1,2]. However, many water bodies are currently under pressure from hydro-morphological alterations, pollution, and over-extraction, which hinder the achievement of good ecological and chemical status [3] as mandated by the European Water Framework Directive [4].

Access to high-quality drinking water is a fundamental human right recognized by the United Nations [5]. Nevertheless, approximately 10% of the global population lives in regions experiencing high or critical water stress [6]. Even in highly developed countries, ensuring sustainable and safe drinking water supplies remains challenging. In the European Union, although 65% of drinking water originates from groundwater, 24% of this resource is chemically compromised and 9% is subject to quantitative limitations [7].

These issues have contributed to a widespread reliance on bottled water, which is predominantly packaged in plastic. Globally, approximately one million plastic bottles are sold every minute [8]. While bottled water remains essential for nearly two billion people lacking access to safe water, its increasing consumption in many developed countries is driven by convenience, portability, perceived health benefits, mistrust of tap water, and personal preference [9]. Conversely, the consumption of properly treated and monitored tap water offers significant advantages, including reduced plastic waste, a lower environmental footprint, and substantial economic savings. For instance, substituting 1.5 L of bottled water with tap water can save approximately 0.34 kg of CO_2_ equivalents [10,11]. Furthermore, the lifecycle of 1 L of bottled water consumes between 17 and 35 L of water [12] and requires up to 2000 times more energy than tap water, excluding the energy costs associated with disposal [13].

Ensuring the safety of tap water requires comprehensive monitoring along the entire supply chain, from water sources and treatment facilities to storage and distribution systems [14]. In Italy, current legislation mandates the monitoring of 53 water quality parameters [15], but plastic particles are not yet included despite growing concern regarding their presence as emerging contaminants [16]. Only recently has the European Union adopted a standardized methodology for detecting microplastics in drinking water (Decision EU 2024/1441) [17].

Numerous studies have reported the presence of plastic particles in drinking water, with concentrations ranging from below detection limits [18,19,20,21,22] to several hundred particles per liter [23]. However, most investigations have focused exclusively on endpoint water samples, such as household taps, without evaluating contamination along the entire drinking water supply chain. This is probably due to the difficulty of accessing the distribution systems of the various drinking water operators. Even comprehensive studies, such as those by Kirstein et al. [24] and Brancaleone et al. [25], either restricted their analysis to distribution systems or focused on geographically dispersed sites not belonging to the same water network.

To address this knowledge gap, the present study provides a comprehensive qualitative and quantitative assessment of plastic particles along an entire drinking water supply chain serving the city of Milan (Northern Italy) to identify whether the plastic contamination at the tap derives from the groundwater source and the pumping station facilities, or whether it is mainly introduced by the distribution network and domestic piping. Samples were collected from groundwater wells, post-treatment stages (activated carbon filtration and accumulation tanks), a public drinking fountain, representing the boundary of the utility’s responsibility, and household taps within ten apartment buildings served by the same distribution network. To the best of our knowledge, this represents the first investigation to evaluate plastic particle occurrence across every stage of a complete water supply infrastructure. Therefore, our dataset could serve as a benchmark for policymakers and stakeholders to more accurately evaluate decisions regarding reference values for plastic contamination in drinking water, as well as to mitigate their presence to ensure enhanced water quality and protect public health.

## 2. Materials and Methods

The monitoring of plastic particles in Milan’s drinking water was conducted prior to the implementation of EU Delegated Decision 2024/1441 [17], which supplements Directive (EU) 2020/2184 [26] by establishing a standardized methodology for the detection of microplastics in drinking water. Consequently, sampling, filtration, and analytical procedures were based on protocols previously described in the scientific literature [27,28,29,30]. It should be noted, however, that our experimental design deviates from this Directive only regarding the minimum sampling volume (1000 L). All other requirements, including the number of replicates, the use of blank samples, and the implementation of robust methods for sampling, separation, and analysis, remain fully aligned with the Directive’s standards. Furthermore, as this study aims to compare plastic contamination in Milan’s distribution system with international data, it was essential to adhere to established methodologies from previous literature, especially given the current lack of data collected under the EU Directive.

### 2.1. Characteristics of Milan’s Water Distribution System

Milan’s drinking water is sourced from the second aquifer, located at depths between 80 and 100 m, through 584 wells, of which approximately 400 are typically active. These wells are connected to 32 distribution stations, with about 28 operating at any given time. The primary treatment process consists of activated carbon filtration [31,32], which removes contaminants such as pesticides and organic solvents through adsorption onto carbon surfaces mediated by van der Waals forces.

After treatment, water is conveyed to an accumulation tank, which functions both as a reservoir and as a sedimentation unit for the removal of suspended particulate matter. Prior to entering the distribution network, the water undergoes mild chlorination.

### 2.2. Sampling

**Figure 1:**
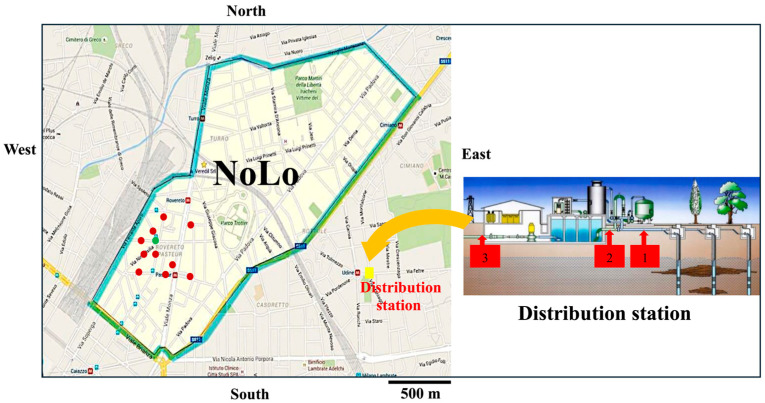
Sampling points from aquifer (n. 1), after activated carbon columns (n. 2), and after the accumulation tank (n. 3) in the distribution station, the fountain (green point) and the 10 buildings (red points) sampled in the NoLo district of Milan.

To assess plastic contamination along the drinking water supply chain, a total of 44 one-liter samples were collected from 14 different sampling points (Figure 1). These included groundwater (five replicates), water collected after activated carbon filtration, after the accumulation tank, from a public drinking water fountain, and from ten household taps located in the NoLo (North of Loreto) district of Milan, all supplied by the same distribution plant (three replicates per sampling point). This neighborhood was chosen because it is completely served by the drinking water distribution station that we were allowed to sample.

Although the Milan aqueduct supplies approximately 7500 L/s [32], the sampled volume was limited to 1 L (in triplicate) due to rapid clogging of the filters by suspended particulate matter during preliminary trials with larger volumes. This sampling volume is consistent with those adopted in comparable studies (Table 1).

All sampling sites were equipped with taps to allow direct collection of water samples. Only the taps in private apartments were equipped with aerator filters, whose visible material was standard metal fixtures or similar. One-liter glass bottles, pre-rinsed with distilled and Milli-Q water (Millipore, Molsheim, France) were used. Before sampling, water was allowed to flow for 30 s to minimize the potential collection of particles accumulated at the tap interface.

To reduce the risk of contamination, operators wore plastic-free clothing, and glass bottles were kept open only for the duration of sample collection. Field blanks were prepared using 8 µm nitrocellulose membrane filters (Sartorius™ (Göttingen, Germany), 50 mm) placed in Petri dishes and subjected to the entire sampling and treatment procedure. Particles detected in blanks that matched those found in samples in terms of shape, color, and polymer type were excluded from the final dataset.

### 2.3. Sample Preparation and Instrumental Analyses

All equipment and glassware were thoroughly rinsed with distilled and Milli-Q water and stored under a laminar flow hood prior to use. Water samples were filtered using a glass vacuum filtration system equipped with 8 µm pore-size nitrocellulose membrane filters [27,28]. After filtration, filters were dried in Petri dishes under laminar flow conditions to prevent airborne contamination. The filtered volume was measured using a graduated glass cylinder.

Suspected plastic particles were visually identified under a stereomicroscope in both samples and blanks, manually isolated using metal tweezers, and transferred onto new membrane filters for subsequent analysis. Polymer identification was performed on individual particles using a Spectrum Two µFTIR spectrometer coupled with a Spotlight 200i microscope (PerkinElmer, Milan, Italy). The limit of detection (LOD) was 20 mm. Spectra were acquired in attenuated total reflectance (ATR) mode using 32 scans over the 600–4000 cm^−1^ range and compared with reference libraries. Only spectra with a match score ≥ 0.70 were considered reliable [27].

Each particle was further characterized by shape (fragments, fibers, films, pellets), color, and size. Particle size was measured using ImageJ software 1.53 K and classified according to ISO/TR 21960:2020 [33] as macroplastics (>5 mm), large microplastics (1–5 mm), microplastics (1 µm–1 mm), and nanoplastics (<1 µm).

**Table 1:** Analytical methods, characteristics, and average concentration of plastic particles detected in water intended for human consumption in various countries worldwide.

Country	Water Source	Sampling Point	Instrumental	Shape	Polymers	Dimension	Plastic Concentration	Sampled Volume	Reference
Denmark	-	taps	FTiR	fibers, fragments, films	PET, PP, PS, other	>10 mm	<LOD	50 L × 3 (10–100 mm); 50 L × 17 (>100 mm)	[18]
Denmark	groundwater	taps	FTiR	Fragments, fibers	PP, PS, PET, other	>10 mm	<LOD	50 L × 3 (10–100 mm); 50 L × 17 (>100 mm)	[19]
Norway	surface water	taps	FTiR	-	-	-	<LOD	1 L × 3 × 24 samples	[20]
Germany	groundwater	taps	FTiR	fibers	PEST, PVC, PE, PA, epoxy resin	50–150 mm	0.7 part./m^3^	40 m^3^	[34]
Germany	groundwater	taps	Raman	-	-	-	<LOQ	0.5–1.5 m^3^	[21]
Germany	groundwater	taps	Raman	-	PE, PET, PP, PA	5–1000 mm	<LOQ	1.3–7.2 m^3^	[22]
Germany	-	taps	FTiR	fragments, fibers, pellets	PS, SEBS, PP, PEST, PE, PVC, other	>19 mm	53 ± 29 part./L	0.5 L × 3	[35]
Finland	-	taps	FTiR	fragments, fibers	PS, SEBS, PP, PEST, PE, PVC, other	>19 mm	47 ± 19 part./L	0.5 L × 3	[35]
France	-	taps	FTiR	fragments, fibers	PS, SEBS, PP, PEST, PE, PVC, other	>19 mm	97 ± 45 part./L	0.5 L × 3	[35]
USA (California and Nevada)	-	taps	FTiR	fragments, fibers, pellets	PS, SEBS, PP, PEST, PE, PVC, other	>19 mm	46 ± 32 part./L	0.5 L × 3	[35]
Mexico	groundwater	fountain	SEM-EDS, Raman	fibers	PEST, epoxy resin	>100 mm	18 ± 7 part./L	1 L × 3 × 42 samples	[36]
China	-	taps	Raman	fragments, fibers, pellets	PE, PP, PE + PP, PPS, PS, PET, other	1–5000 mm	440 ± 275 part./L	1 L × 38 samples	[23]
China	surface water	taps	SEM, FTiR, Raman	fragments, fibers, pellets	PA, PVC, PP, PET, PE, other	1–10 mm; 10–100 mm; >100 mm	266 ± 56 part./L 63 ± 11 part./L 14 ± 5 part./L	10 L × 3 × 4 samples	[37]
China	groundwater	taps	FTiR	fragments, fibers	PEST, PA, PS	>10 mm	13.23 part./L	1 L × 2	[38]
Japan	groundwater and surface water	taps	FTiR	fragments, fibers, pellets	PS, SEBS, PP, PEST, PE, PVC, other	>19 mm	29 ± 45 part./L	0.5 L × 28 samples	[35]
Saudi Arabia	desalted water	taps	FTiR	-	PE	25–500 mm	1.8 part./L	1 L	[39]
Sweden (Skåne)	groundwater	aquifer, supply pipelines	mFTiR-Py-GCMS	fragments, fibers	PEST, PA, PE, PVC, PS, PU, PP, acrylic	5.2–374 mm	174 ± 405 part./m^3^ (average)	200–1100 L × 3	[24]
Italy (Lazio)	groundwater	aquifer plant outlet water kiosks fountains taps glass and plastic bottles	m-Raman	fragments, fibers, pellets	PTFE, PP, PET, PE	30–100 mm	5.0 ± 1.5 part./L <1 part./L <LOQ 5 ± 1.5 part./L 2 ± 1 part./L <LOQ	1 L × 34 samples	[25]
Italy (Milan)	groundwater	aquifer carbon filters accum. tank fountain taps	mFTiR	fragments, fibers, pellets, films	PEST, PAK, PTFE, PP, PU, PA, ABS, PS	30–3600 mm	0.9 ± 1.1 part./L 2.0 ± 2.8 part./L 0.3 ± 0.5 part./L 1.0 ± 0.0 part./L 1.9 ± 1.4 part./L	1 L × 3 × 14 samples	Present study

### 2.4. Statistical Analysis

The statistical robustness of the study was ensured by a multi-step analytical approach. The Kolmogorov–Smirnov and Bartlett’s tests were employed to confirm that the data followed a normal distribution and maintained homogeneity of variance, fulfilling the necessary assumptions for parametric analysis. Subsequently, a one-way ANOVA was conducted to determine whether significant differences in plastic particle concentrations existed across the various sampling points. To further pinpoint specific variations between individual points, Tukey’s post hoc test was applied. All results were evaluated at a significance threshold of *p* < 0.05.

## 3. Results

Very few plastic particles were detected in the blank filters, as detailed in Table S1. Specifically, a single transparent polyester fiber (PEST) was found in the blank filters used for the groundwater, activated carbon filters, and accumulation tank samples, respectively. Additionally, one red polyurethane (PU) film was identified in the blank for Apartment 1, two transparent PEST fibers in Apartment 4, and one transparent PEST fiber in Apartment 8. No other plastic particles from the external environment were observed in the remaining blank filters.

### 3.1. Quantitative Characterization

Table S2 summarizes the characteristics of the 863 particles initially identified via stereomicroscopy as potential plastics. Subsequent μFT-IR analysis confirmed that only 73 of these particles (8%) were actually plastic. The majority (76%) were cellulose-based, while the remaining 16% consisted of inorganic materials (e.g., calcium carbonate, quartz, and various silicates/sulfates) with match scores consistently below 0.70.

**Figure 2:**
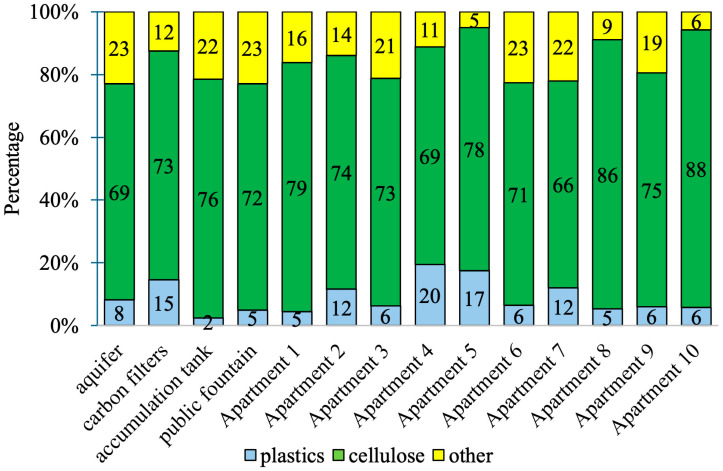
Percent composition of particles detected in samples from individual sampling points.

The proportion of confirmed plastic particles varied substantially across the 14 sampling sites, ranging from 2% in the accumulation tank to 20% in tap water from Apartment 4 (Figure 2). Within the distribution station, plastic particles accounted for 15% of the total downstream of the activated carbon filters, 8% in the groundwater, and 2% in the accumulation tank.

**Figure 3:**
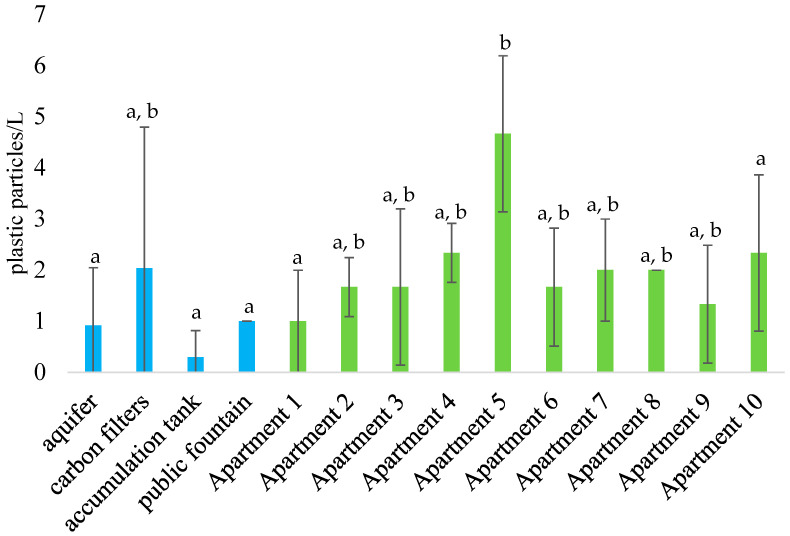
Mean concentration of plastic particles detected along the monitored drinking water supply chain. Sampling points under water utility management are shown in blue; those from private apartments are shown in green. Different letter points out significant differences (*p* < 0.05) between sampling points. Replicates for aquifer were *n* = 5, while for all the other sampling points, *n* = 3.

When focusing exclusively on plastic particles, concentrations showed notable variations (Figure 3). Inside the distribution station, values ranged from 0.3 ± 0.5 particles/L (post-accumulation tank) to 2.0 ± 2.8 particles/L (post-activated carbon filtration); however, these differences were not statistically significant (*p* > 0.05). In the private apartments, concentrations ranged from 1.0 ± 1.0 particles/L (Apartment 1) to a peak of 4.7 ± 1.5 particles/L (Apartment 5), with the latter being significantly higher than several other sampling points (Figure 3). The overall mean concentration for private apartments was 1.9 ± 1.4 particles/L, which did not differ significantly from the levels measured at the distribution station.

### 3.2. Qualitative Characterization

**Figure 4:**
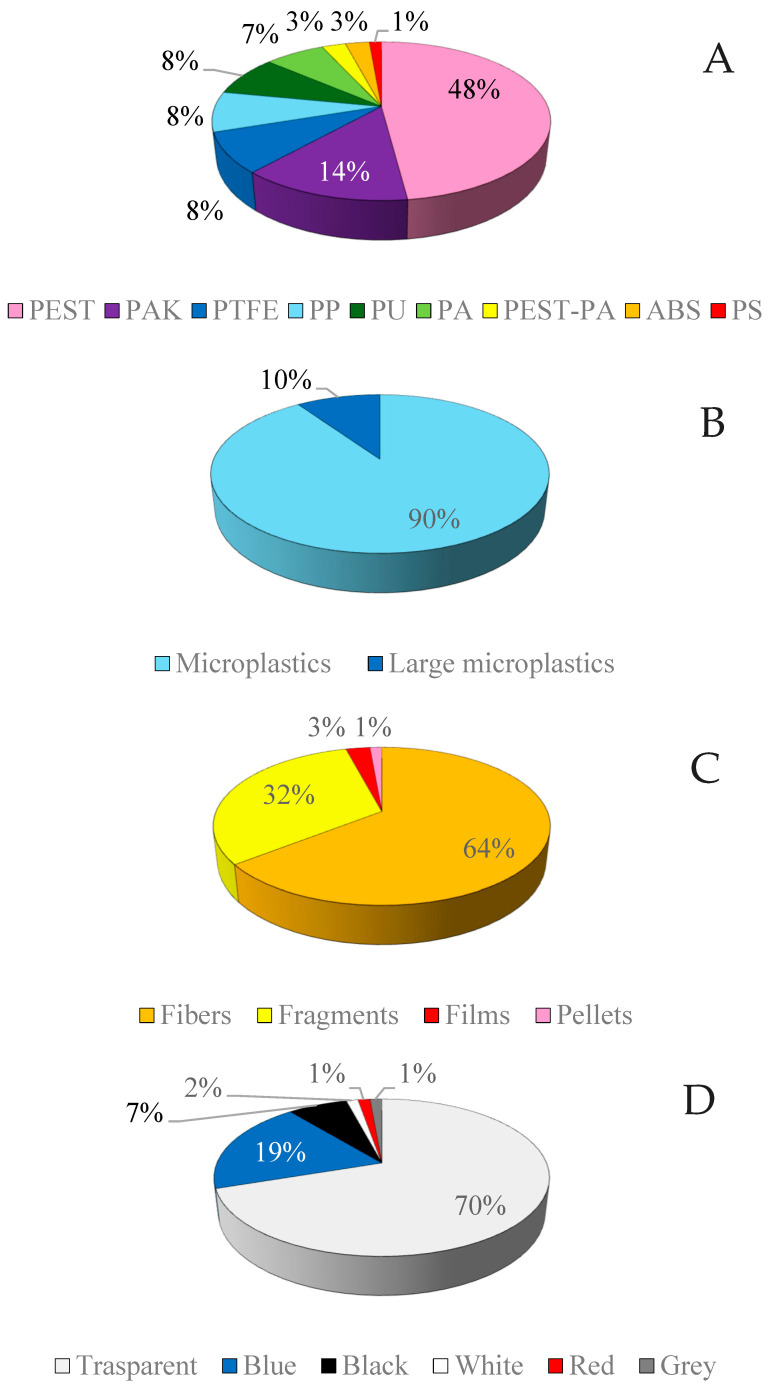
Characteristics of the complete dataset of plastic particles monitored at the 14 sampling points. (**A**) Percentage distribution of polymer types; (**B**) Percentage distribution by particle size; (**C**) Percentage distribution by particle shape; (**D**) Percentage distribution by particle color. PEST = polyester; PAK = polyacrylate; PTFE = polytetrafluoroethylene; PP = polypropylene; PU = polyurethane; PA = polyamide; ABS = acrylonitrile-butadiene-styrene; PS = polystyrene.

The 73 identified plastic particles were characterized by polymer type, size, shape, and color. PEST was the most prevalent polymer (48%), followed by polyacrylate (PAK, 14%), polytetrafluoroethylene (PTFE, 8%), polypropylene (PP, 8%), and PU (8%). Other polymers detected less frequently included polyamide (PA, 7%), the PEST-PA co-polymer (3%), acrylonitrile-butadiene-styrene (ABS, 3%), and polystyrene (PS, 1%) (Figure 4A).

In terms of size, the vast majority (90%) were microplastics (MPs), while 10% were classified as large microplastics (LMPs) (Figure 4B). Fibers were the dominant shape (64%), followed by fragments (32%), whereas films (3%) and pellets (1%) were rare (Figure 4C). Color analysis showed that 70% of the particles were transparent, followed by blue (19%) and black (7%), with minor percentages of white, red, and gray (Figure 4D).

**Figure 5:**
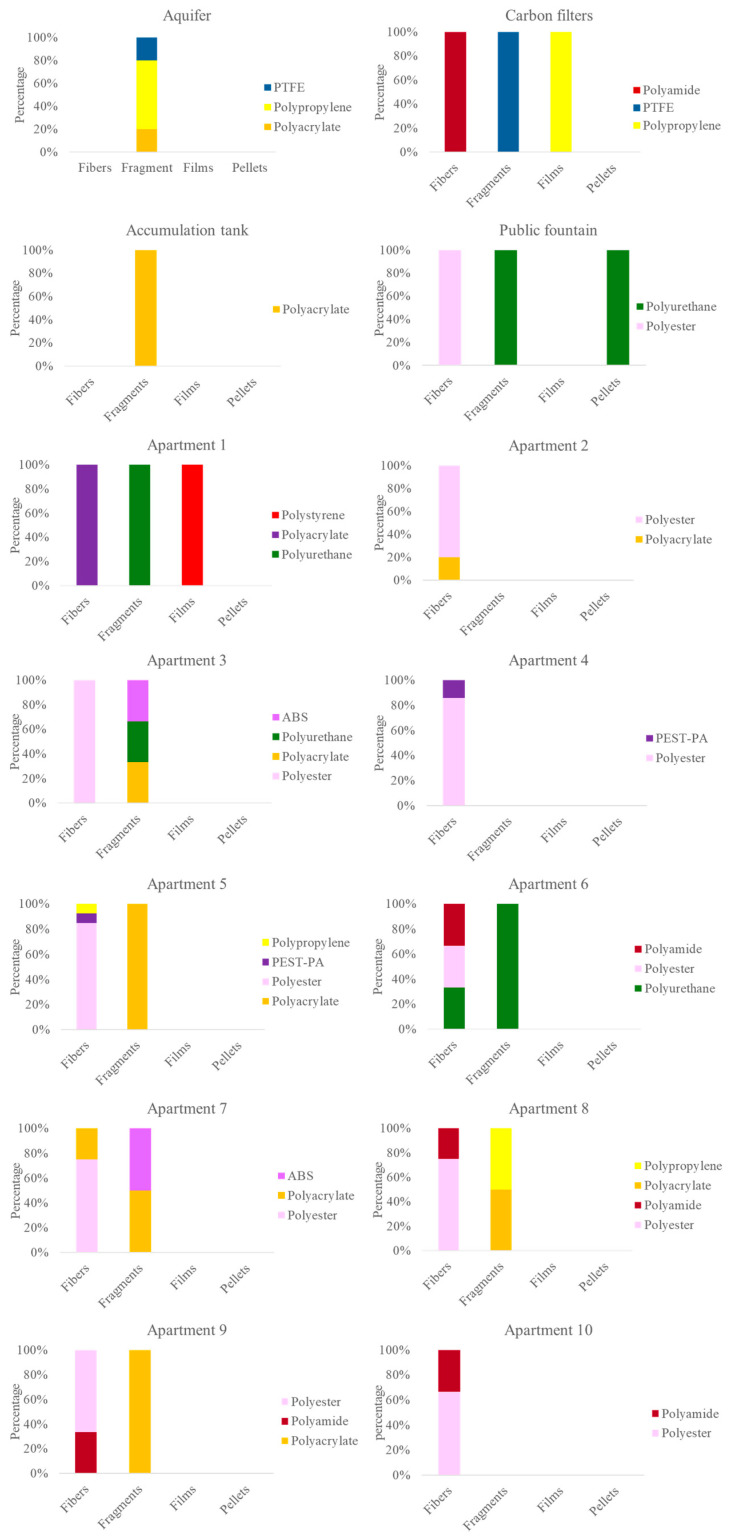
Polymeric composition of each shape class identified for the individual sampling points.

A highly informative analysis of the contamination characteristics and their potential origins is presented in Figure 5, which integrates data on particle shape and polymer composition. Overall, all particles identified as PEST, PA, and PEST-PA were fibers, whereas those classified as PTFE and ABS were exclusively fragments. More specifically, 75% of all detected fibers were composed of PEST, followed by PA (11%) and PAK (6%), with the remaining 8% consisting of other identified polymers. Among the fragments, 31% were composed of PAK, followed by PTFE (26%), PP and PU (17% each), and the ABS copolymer (9%) (Figure 5). Additionally, the two detected films were composed of PP and PS, respectively, while the only identified pellet consisted of PU. Overall, all the particles identified as PEST, PA, and PEST-PA were fibers, whereas those classified as PTFE and ABS were exclusively fragments. More specifically, 75% of all detected fibers were composed of PEST, followed by PA (11%) and PAK (6%), while the remaining 8% consisted of other identified polymers. Among the fragments, 31% were made of PAK, followed by PTFE (26%), PP and PU (17% each), and the ABS copolymer (9%) (Figure 5). Additionally, the two detected films were composed of PP and PS, respectively, and the only identified pellet was made of PU.

Given the wide variability observed in the characteristics of the examined plastic particles, a more detailed description of their distribution across the various sampling points along the drinking water supply chain is warranted. Indeed, both particle shape and polymer composition varied within the distribution station. Only fragments were detected in the groundwater and the accumulation tank, whereas fibers and films emerged downstream of the activated carbon filters. Polymer composition also shifted: PAK was present in the groundwater and the accumulation tank but was absent after carbon filtration. In contrast, PA fibers appeared only after the carbon filters. Greater heterogeneity was observed in the apartment samples. Some (Apartments 2, 4, and 10) contained mainly PEST fibers, while others (Apartment 1) exhibited more complex compositions, including the only film found in any private sample. Notably, Apartment 1 was the only household sample where PEST fibers were absent. Figures S1–S3 present the data obtained for each sampling point regarding the shape, size, and color of the examined plastic particles, respectively.

## 4. Discussion

### 4.1. Characterization of Plastic Contamination Along the Drinking Water Supply Chain

A primary finding that warrants immediate emphasis is the discrepancy between visual inspection and spectroscopic confirmation: on average, only 8% of visually pre-selected particles were identified as plastic by μFTIR analysis. This result underscores the critical necessity of employing advanced instrumental techniques, as relying solely on visual sorting leads to a significant overestimation of plastic contamination. Consequently, these findings highlight that instrumental validation is indispensable for generating accurate, high-quality data and for establishing a reliable baseline in drinking water research.

Based on the data presented above, plastic particles were detected at all sampling points, as also shown in Table S2. This confirms that the volume of water collected at each site was sufficient to provide a reliable overview of contamination trends along Milan’s drinking water supply chain. It should be noted, however, that the small sampling volumes (5 L for groundwater and 3 L for all other sampling points) may lead to an underestimation of rare polymers and sporadic events of contamination due to the heterogeneous distribution of such emerging contaminants.

The lowest concentrations were measured in the aquifer and the water exiting the accumulation tank, while the highest averages were observed in the water post-carbon filtration and within the ten private apartments (Table 2). Nonetheless, no statistically significant differences were observed among the average values across the distribution system.

The identified characteristics of the plastic particles allow for the formulation of hypotheses regarding potential contamination sources. For instance, despite the very low concentrations detected in groundwater, it is crucial to elucidate its provenance. This may be attributed to pipes, pumps, and machinery utilized for aquifer extraction [40], or to direct terrestrial contamination infiltrating the drinking water source. The migration of plastic particles in groundwater is an exceedingly complex phenomenon, influenced by factors such as aquifer recharge timing, soil porosity, and the physicochemical properties of the plastics [41]. While Milan’s aquifers are primarily recharged by precipitation and surface waters (rivers and canals) [42], where plastics have been previously documented [28,43], identifying a definitive origin remains challenging. However, we can hypothesize on the prevalence of these sources based on the fact that only fragments of three specific polymers (PAK, PP, and PTFE) were identified in the aquifer. The latter two are widely used in water infrastructure components, such as pipes, gaskets, and valves [25,44,45]. Thus, the fragmentation of these components likely represents an indirect source of contamination. Furthermore, Milan’s drinking water aquifer lies at a considerable depth (80–100 m), and the subsoil is predominantly composed of silt and clay [46]. Given that the pores in such soil range from 0.01 to 0.05 µm, and the smallest fragment detected in the aquifer measured 0.21 mm, terrestrial infiltration is highly improbable. Consequently, we hypothesize that these fragments result from the degradation of extraction machinery rather than terrestrial sources, although the latter cannot be entirely ruled out. Evidently, these hypotheses must be subsequently validated through further studies focused exclusively on assessing the origin of groundwater contamination.

Notably, water exiting the activated carbon filters exhibited higher contamination levels than both the groundwater and the accumulation tank. The efficacy of such filters is a subject of debate: some studies report a ~60% reduction in plastic particles [47,48,49], while others have observed an increase, likely due to pre-existing contamination of the carbon granules and the subsequent release of plastics due to water flow and/or mechanical abrasion of carbon granules [50] or saturation leading to the release of previously adsorbed particles [51]. Our results support this second scenario, as detected levels were twice as high as those in the aquifer and nearly seven times higher than those in the accumulation tank (Table 2). Conversely, the accumulation tank appears to be an excellent removal system, likely due to sedimentation. This is consistent with recent findings [52] suggesting that approximately 45% of plastic particles can be removed through sedimentation, a process governed by particle size and density [52,53,54]. Our data show that the accumulation tank effectively removed almost all particles exiting the filters, with the exception of one small PAK fragment (0.03 mm). Its detection is likely explained by its small size and a density (1.0511 g/cm^3^) very close to that of water, which hindered sedimentation.

Moving downstream from the treatment plant, water sampled from the public fountain, which marks the boundary of the utility provider’s responsibility, exhibited a slightly higher, though not significantly different, concentration compared to the water exiting the accumulation tank (Table 2). Specifically, one PEST fiber, one PU fragment, and one PU pellet were identified; notably, none of these polymers were detected in samples collected within the distribution station (Figure 5). Since Milan’s main pipelines are composed of steel (19%) and cast iron (81%), with service connections made of PE [31,32], a polymer not identified at any prior stage of the supply chain, it is evident that the source of these particles cannot be attributed to the infrastructure components. The most plausible source of plastic contamination in the pipelines downstream of the plant is atmospheric fallout [35,55,56]. This may occur as air pockets form within the distribution network during pipe drainage, particularly in areas with intermittent supply, or following maintenance and pipe replacement activities conducted by the water provider.

Plastic concentrations increased in water collected from the taps of the ten private apartments served by the same distribution plant, reaching an average of 1.9 ± 1.4 particles/L (Table 2). Levels peaked at 4.7 ± 1.5 particles/L in Apartment 5, a value significantly higher than those found at many other sampling points along the supply chain (Figure 3). Despite the substantial quantitative and qualitative variability among the apartment samples, several common factors support a hypothesis regarding the origin of this contamination. For instance, polymers widely used in water distribution components [25,34,45], such as ABS, PE, PA, and PP, were detected in seven out of the ten apartments (Figure 5). Similarly, PU, which is commonly employed for thermal insulation, waterproof coatings for cisterns, joint sealants, and pipe repairs, was identified in these samples despite being absent within the distribution plant (Figure 5). Furthermore, residential buildings often utilize water storage tanks or pressure booster systems typically constructed from plastic materials (PE, PP, ABS, PVC), representing a potential source of particle release into the internal plumbing [57,58]. However, the detection of numerous PEST and PA fibers, which were absent within the distribution plant and are typically associated with atmospheric deposition [35,55,56], suggests a dual origin for plastic contamination in private dwellings. This contamination likely stems not only from the degradation of internal pipes and storage tanks but also from external infiltration. For example, ‘hidden leaks’, water leaks occurring downstream of the meter within walls or underground, are not directly visible or inspectable. Such structural failures in the private section of the system can serve as entry points for external plastic particles into the drinking water supply. Contamination of the sample by these synthetic fibers from the sampled domestic environments can be ruled out, both due to the use of a blank for each sampling point and the care taken by the operator to wear non-synthetic clothing. Furthermore, the mouth of the sampling bottles was only 26 mm wide, and sampling took no longer than a couple of minutes. However, these hypotheses regarding the origin of the contamination will also need to be confirmed in subsequent studies.

In conclusion, while plastic contamination in the groundwater and treatment plant remains remarkably low, building-specific piping and storage systems appear to contribute to the increased levels observed at the tap.

### 4.2. Comparison of Plastic Levels Along Drinking Water Supply Chains Worldwide

Comparing results across previous studies assessing plastic concentrations in water intended for human consumption is hindered by the lack of uniform criteria for size classification, as well as the absence of standardized protocols for sampling, treatment, and instrumental identification. Table 1 summarizes the plastic concentrations detected at various points along drinking water supply chains globally. As observed, most research has focused on household tap water, with only one study considering samples from a public fountain [36]. To the best of our knowledge, besides the present work, only two investigations [24,25] have evaluated plastic occurrence at multiple stages of the supply chain, albeit with the limitations previously noted (see Introduction). Another commonality among studies is the sampling volume, typically ranging from 0.5 to 1 L per sample, consistent with our experimental design of triplicate (or fivefold for groundwater) sampling. While other studies [21,22,34] employed larger volumes, they rarely exceeded 1 m^3^, the threshold recently established by EU Delegated Decision 2024/1441 [17]. Finally, infrared and Raman spectroscopy remain the most widely adopted instrumental techniques (Table 1).

Regarding the comparison with existing data, plastic particle levels in drinking water vary considerably regardless of the source. Specifically, the average concentration detected in Milan’s tap water (1.9 ± 1.4 particles/L) is substantially lower than levels reported in several European surveys [35], where concentrations ranged from 47 ± 19 particles/L (Finland) to 97 ± 45 particles/L (France). It is also markedly lower than values from global campaigns [23,35,37,38], which reached up to 440 ± 275 particles/L in China. Conversely, several studies, particularly from Northern Europe, have reported negligible concentrations, such as 0.7 particles/m^3^ in Germany [34], or levels below the limit of detection (LOD) [18,19,20] or quantification (LOQ) [20,22]. Nevertheless, the European average is estimated at 3.6 particles/L [59]. The low level reported in Saudi Arabia (1.8 particles/L) [39] is nearly identical to our findings but was measured in desalinated water, a matrix subjected to specific treatments like nanofiltration and reverse osmosis, which may inherently reduce plastic content.

A distinct comparison can be made with the only two studies that analyzed multiple segments of the supply chain. The average plastic concentration (174 ± 405 particles/m^3^) detected in Swedish groundwater and two distribution pipelines [24] corresponds to approximately 0.2 ± 0.4 particles/L, which is fully comparable to our observations at the distribution plant outlet (0.3 ± 0.5 particles/L). Furthermore, data from a large-scale supply chain in Central Italy (Lazio) showed values closely aligned with ours: slightly higher concentrations in groundwater and public fountains (5.0 ± 1.5 particles/L) but identical average levels in tap water (2.0 ± 1.0 particles/L) [25].

In conclusion, the water sampled along Milan’s drinking water supply chain exhibits low levels of plastic contamination, placing it within the lowest ranges observed worldwide, even when accounting for the analytical and instrumental differences across studies. It is possible that this low level of plastic contamination, particularly at the distribution station, is due to the abstraction of water from Milan’s deep groundwater aquifer. Such high quality may not be guaranteed when water intended for human consumption is sourced from surface waters.

### 4.3. Human Exposure Assessment

The primary pathways for human exposure to plastic particles are ingestion and inhalation [60]. While the skin acts as a barrier to particles larger than 100 nm, translocation may still occur via hair follicles, sweat glands, or dermal lesions [61]. Several studies [60,62,63,64] have estimated human exposure by assessing plastic intake through various foods and beverages; however, a direct comparison of these data remains challenging due to the lack of standardized analytical and instrumental methodologies.

A recent study [64] calculated both the average number of MPs ingested (ANMP) and their cumulative mass. This research estimated an ANMP ranging from 19,085 to 86,576 particles/person/year, based on tap water consumption (16,265–68,331 particles/year) and three other consumable categories with sufficient data (shellfish, salt, and beer). These findings suggest that water consumption is the predominant source of plastic ingestion, accounting for over 80% of the total estimated theoretical intake.

By contextualizing these levels with our findings and considering the Italian Adequate Intake (AI) for adults over 18 (2.5 L/day for males and 2.0 L/day for females [65]), new exposure metrics emerge. Based on the average concentration detected in the evaluated supply system (1.9 particles/L), an adult male would ingest approximately 1734 plastic particles/year, while an adult female would ingest around 1387 particles/year through exclusive tap water consumption. These values are substantially lower than those reported in previous study, representing only 2.5–11.0% (males) and 2.0–8.5% (females) of the tap-water-specific ANMP estimated by Senathirajah et al. [64]. On a broader scale, these quantities account for only 2.0–9.0% and 1.6–7.0% of the total estimated annual plastic intake for males and females, respectively. These results underscore that tap water consumption from the supply system evaluated in this study contributes only minimally to overall human exposure to plastic particles.

## 5. Conclusions

This study represents the first investigation to monitor plastic contamination throughout an entire drinking water supply chain, from the aquifer to the household tap. As such, it provides a crucial benchmark for future research, the development of regulatory guidelines, and the strategic management of drinking water resources. This is particularly relevant in countries like Italy, where approximately 85% of drinking water is sourced from groundwater.

Our findings demonstrate that while plastic particle concentrations in the investigated aquifer are remarkably low, there is a noticeable increase in contamination within private building plumbing systems. These results suggest that internal distribution networks are a probable key source of contamination. Consequently, they should therefore be subject to enhanced monitoring and more rigorous inspection, especially in anticipation of future regulations that may establish maximum allowable limits for plastic content in drinking water.

Future research should expand this comprehensive monitoring approach to diverse urban water systems and seasons to evaluate how different geological contexts, groundwater depth and infrastructure types might influence plastic occurrence. Furthermore, long-term studies are essential to assess the degradation rates of private plumbing materials and their specific contribution to domestic contamination.

Despite the variability observed in private apartments, the concentrations measured in this study remain among the lowest reported worldwide. From a public health perspective, the estimated exposure through this supply system represents only a minor fraction of the total annual plastic intake from all sources. However, a limitation of the current instrumental approach is its inability to detect potential nanoplastic contamination, a task that remains a critical frontier for future drinking water monitoring.

## Figures and Tables

**Table 2:** Mean concentration of plastic particles detected in samples from various positions along the drinking water supply chain.

Sampling Point	Mean Concentration (Particles ± St. Dev./L)	Min Value (Particles/L)	Max Value (Particles/L)
aquifer	0.9 ± 1.1	0.0	2.8
carbon filters	2.0 ± 2.8	0.0	5.2
accumulation tank	0.3 ± 0.5	0.0	0.9
public fountain	1.0 ± 0.0	1.0	1.0
apartments 1–10	1.9 ± 1.4	0.0	6.0

## Data Availability

The original contributions presented in this study are included in the article/Supplementary Materials. Further inquiries can be directed to the corresponding authors.

## Supplementary Materials

The following supporting information can be downloaded at: https://www.mdpi.com/article/10.3390/jox16010018/s1, Figure S1: Shape-based classification of plastic particles detected in samples from individual sampling points; Figure S2: Size-based classification of plastic particles detected in samples from individual sampling points; Figure S3: Color-based classification of plastic particles detected in samples from individual sampling points; Table S1: Detected particles in blank samples; Table S2: Particles detected in the 44 samples.

## References

[1] World Health Organization (WHO) (2017). Guidelines for Drinking-Water Quality: Fourth Edition Incorporating the First Addendum.

[2] United Nations (UN) (2021). The United Nations World Water Development Report 2021: Valuing Water.

[3] European Environment Agency (EEA) (2024). Europe’s State of Water 2024: The Need for Improved Water Resilience.

[4] European Environmental Agency (EEA) (2021). Drivers of and Pressures Arising from Selected Key Water Management Challenges—A European Overview.

[5] United Nations (UN) (2016). The Human Rights to Safe Drinking Water and Sanitation: Resolution Adopted by the General Assembly.

[6] United Nations (UN) (2023). The United Nations World Water Development Report 2023: Partnerships and Cooperation for Water.

[7] European Environmental Agency (EEA) (2022). Europe’s Groundwater—A Key Resource Under Pressure.

[8] Abraham A., Cheema S., Chaabna K., Lowenfels A.B., Mamtani R. (2024). Rethinking bottled water in public health discourse. BMJ Glob. Health.

[9] Tosun J., Scherer U., Schaub S., Horn H. (2020). Making Europe go from bottles to the tap: Political and societal attempts to induce behavioral change. Wiley Interdiscip. Rev.-Water.

[10] Galati A., Alaimo L.S., Ciaccio T., Vrontis D., Fiore M. (2022). Plastic or not plastic? That’s the problem: Analysing the Italian students purchasing behavior of mineral water bottles made with eco-friendly packaging. Resour. Conserv. Recycl..

[11] Amicarelli V., Bux C., Lagioia G., Lagioia G., Paiano A., Amicarelli V., Gallucci T., Ingrao C. (2024). Environmental Accounting for the Circularization of the Packaged Water Sector in Italy. Innovation, Quality and Sustainability for a Resilient Circular Economy Springer.

[12] Parag Y., Elimelech E., Opher T. (2023). Bottled Water: An Evidence-Based Overview of Economic Viability, Environmental Impact, and Social Equity. Sustainability.

[13] Geerts R., Vandermoere F., Van Winckel T., Halet D., Joos P., Van Den Steen K., Van Meenen E., Blust R., Borregán-Ochando E., Vlaeminck S.E. (2020). Bottle or tap? Toward an integrated approach to water type consumption. Water Res..

[14] World Health Organization (WHO) (2011). Guidelines for Drinking-Water Quality.

[15] Legislative Decree (18/2023). http://www.gazzettaufficiale.it/eli/id/2023/03/06/23G00025/sg.

[16] Swain P.R., Parida P.K., Majhi P.J., Behera B.K., Das B.K. (2025). Microplastics as Emerging Contaminants: Challenges in Inland Aquatic Food Web. Water.

[17] Decision EU 2024/1441. https://eur-lex.europa.eu/eli/dec_del/2024/1441/oj/eng.

[18] Strand J., Feld L., Murphy F., Mackevica A., Hartmann N.B. (2018). Analysis of Microplastic Particles in Danish Drinking Water.

[19] Feld L., Silva V.H.D., Murphy F., Hartmann N.B., Strand J. (2021). A study of microplastic particles in Danish tap water. Water.

[20] Uhl W., Eftekhardadkhah M., Svendsen C. (2018). Mapping microplastic in Norwegian drinking water. Atlantic.

[21] Weber F., Kerpen J., Wolff S., Langer R., Eschweiler V. (2021). Investigation of microplastics contamination in drinking water of a German city. Sci. Total Environ..

[22] Pittroff M., Müller Y.K., Witzig C.S., Scheurer M., Storck F.R., Zumbülte N. (2021). Microplastic analysis in drinking water based on fractionated filtration sampling and Raman microspectroscopy. Environ. Sci. Pollut. Res..

[23] Tong H., Jiang Q., Hu X., Zhong X. (2020). Occurrence and identification of microplastics in tap water from China. Chemosphere.

[24] Kirstein I.V., Hensel F., Gomiero A., Iordachescu L., Vianello A., Wittgren H.B., Vollertsen J. (2021). Drinking plastics?—Quantification and qualification of microplastics in drinking water distribution systems by μFTIR and Py-GCMS. Water Res..

[25] Brancaleone E., Mattei D., Fuscoletti V., Lucentini L., Favero G., Cecchini G., Frugis A., Gioia V., Lazzazzara M. (2024). Microplastic in Drinking Water: A Pilot Study. Microplastics.

[26] Directive (EU) 2020/2184. https://eur-lex.europa.eu/eli/dir/2020/2184/oj/eng.

[27] Magni S., Nigro L., Della Torre C., Binelli A. (2021). Characterization of plastics and their ecotoxicological effects in the Lambro River (N. Italy). J. Hazard. Mater..

[28] Magni S., Della Torre C., Nigro L., Binelli A. (2022). Can COVID-19 pandemic change plastic contamination? The Case study of seven watercourses in the metropolitan city of Milan (N. Italy). Sci. Total Environ..

[29] Binelli A., Magni S., Della Torre C., Sbarberi R., Cremonesi C., Galafassi S. (2024). Monthly variability of floating plastic contamination in Lake Maggiore (Northern Italy). Sci. Total Environ..

[30] Binelli A., Tognetto M., Cremonesi C., Della Torre C., Caorsi G., Magni S. (2025). Dietary exposure and risk assessment of plastic particles in cow’s milk stored in various packaging materials. J. Hazard. Mater..

[31] https://www.centraleacquamilano.it/.

[32] https://latuacqua.it/.

[33] (2020). Plastics—Environmental Aspects—State of Knowledge and Methodologies.

[34] Mintenig S.M., Löder M.G., Primpke S., Gerdts G. (2019). Low numbers of microplastics detected in drinking water from ground water sources. Sci. Total Environ..

[35] Mukotaka A., Kataoka T., Nihei Y. (2021). Rapid analytical method for characterization and quantification of microplastics in tap water using a Fourier-transform infrared microscope. Sci. Total Environ..

[36] Shruti V.C., Pérez-Guevara F., Kutralam-Muniasamy G. (2020). Metro station free drinking water fountain-A potential “microplastics hotspot” for human consumption. Environ. Pollut..

[37] Shen M., Zeng Z., Wen X., Ren X., Zeng G., Zhang Y., Xiao R. (2021). Presence of microplastics in drinking water from freshwater sources: The investigation in Changsha, China. Environ. Sci. Pollut. Res..

[38] Chu X., Zheng B., Li Z., Cai C., Peng Z., Zhao P., Tian Y. (2022). Occurrence and distribution of microplastics in water supply systems: In water and pipe scales. Sci. Total Environ..

[39] Almaiman L., Aljomah A., Bineid M., Aljejdh F.M., Aldawsari F., Liebmann B., Lomako I., Sexlinger K., Alarfaj R. (2021). The occurrence and dietary intake related to the presence of microplastics in drinking water in Saudi Arabia. Environ. Monit. Assess..

[40] Viaroli S., Lancia M., Lee J., Ben Y., Giannecchini R., Castelvetro V., Petrini R., Zheng C., Re V. (2024). Limits, challenges, and opportunities of sampling groundwater wells with plastic casings for microplastic investigations. Sci. Total Environ..

[41] Severini E., Ducci L., Sutti A., Robottom S., Sutti S., Celico F. (2022). River–groundwater interaction and recharge effects on microplastics contamination of groundwater in confined alluvial aquifers. Water.

[42] Romano E., Arduini C., Belli A., Carminati A., di Palma F., Giudici M., Piazzolla D., Villa D. (2002). A large scale model of ground water flow in Milano (Italy). Geophysical Research Abstracts.

[43] Binelli A., Della Torre C., Nigro L., Riccardi N., Magni S. (2022). A realistic approach for the assessment of plastic contamination and its ecotoxicological consequences: A case study in the metropolitan city of Milan (N. Italy). Sci. Total Environ..

[44] Dhanumalayan E., Joshi G.M. (2018). Performance properties and applications of polytetrafluoroethylene (PTFE)—A review. Adv. Compos. Hybrid Mater..

[45] Espejo E. (2023). Analysis of Premature Failure of a PVC Water Pipe. J. Fail. Anal. Prev..

[46] Marzorati A. (2014). Analisi Dell’influenza Delle Gallerie Metropolitane Sull’innalzamento del Livello di Falda: Il Caso di Milano. Master’s Thesis.

[47] Pivokonsky M., Cermakova L., Novotna K., Peer P., Cajthaml T., Janda V. (2018). Occurrence of microplastics in raw and treated drinking water. Sci. Total Environ..

[48] Pivokonsky M., Pivokonská L., Novotná K., Čermáková L., Klimtová M. (2020). Occurrence and fate of microplastics at two different drinking water treatment plants within a river catchment. Sci. Total Environ..

[49] Wang Z., Lin T., Chen W. (2020). Occurrence and removal of microplastics in an advanced drinking water treatment plant (ADWTP). Sci. Total Environ..

[50] Bäuerlein P.S., Hofman-Caris R.C.H.M., Pieke E.N., ter Laak T.L. (2022). Fate of microplastics in the drinking water production. Water Res..

[51] Pillai S.B., Lahnsteiner J. (2020). Adsorption in water and used water purification. Handbook of Water and Used Water Purification.

[52] Li Y., Meng Y., Qin L., Shen M., Qin T., Chen X., Chai B., Liu Y., Dou Y., Duan X. (2024). Occurrence and removal efficiency of microplastics in four drinking water treatment plants in Zhengzhou, China. Water.

[53] Tursi A., Baratta M., Easton T., Chatzisymeon E., Chidichimo F., De Biase M., De Filpo G. (2022). Microplastics in aquatic systems, a comprehensive review: Origination, accumulation, impact, and removal technologies. RSC Adv..

[54] Sacco N.A., Zoppas F.M., Devard A., González Muñoz M.D.P., García G., Marchesini F.A. (2023). Recent advances in microplastics removal from water with special attention given to photocatalytic degradation: Review of scientific research. Microplastics.

[55] Zhang Y., Kang S., Allen S., Allen D., Gao T., Sillanpää M. (2020). Atmospheric microplastics: A review on current status and perspectives. Earth-Sci. Rev..

[56] Gambino I., Bagordo F., Grassi T., Panico A., De Donno A. (2022). Occurrence of microplastics in tap and bottled water: Current knowledge. Int. J. Environ. Res. Public Health.

[57] Acarer S. (2023). Abundance and characteristics of microplastics in drinking water treatment plants, distribution systems, water from refill kiosks, tap waters and bottled waters. Sci. Total Environ..

[58] Cao N.-D., Vo D.-H.T., Pham M.-D., Nguyen V.-T., Nguyen T.-B., Le L.-T., Mukhtar H., Nguyen H.-V., Visvanathan C., Bui X.-T. (2024). Microplastics contamination in water supply system and treatment processes. Sci. Total Environ..

[59] Kosuth M., Mason S.A., Wattenberg E.V. (2018). Anthropogenic contamination of tap water, beer, and sea salt. PLoS ONE.

[60] Zuri G., Karanasiou A., Lacorte S. (2023). Microplastics: Human exposure assessment through air, water, and food. Environ. Internat..

[61] Ageel H.K., Harrad S., Abdallah M.A.E. (2022). Occurrence, human exposure, and risk of microplastics in the indoor environment. Environ. Sci. Process. Impacts.

[62] Cox K.D., Covernton G.A., Davies H.L., Dower J.F., Juanes F., Dudas S.E. (2019). Human Consumption of Microplastics. Environ. Sci. Technol..

[63] Zhang Q., Xu E.G., Li J., Chen Q., Ma L., Zeng E.Y., Shi H. (2020). A review of microplastics in table salt, drinking water, and air: Direct human exposure. Environ. Sci. Technol..

[64] Senathirajah K., Attwood S., Bhagwat G., Carbery M., Wilson S., Palanisami T. (2021). Estimation of the mass of microplastics ingested–A pivotal first step towards human health risk assessment. J. Hazard. Mater..

[65] SINU, Società Italiana di Nutrizione Umana (2024). Livelli di Assunzione di Riferimento di Nutrienti ed Energia per la Popolazione Italiana. V Versione.

