# Supplementary material for: From Aquifer to Tap: Comprehensive Quali-Quantitative Evaluation of Plastic Particles Along a Drinking Water Supply Chain of Milan (Northern Italy)
Source: J Xenobiot. 2026 Jan 22;16(1):18. doi: 10.3390/jox16010018 (PMC12921940; doi:10.3390/jox16010018)
Supplement: Supplementary file 1 [file jox-16-00018-s001.zip › Figure S1.pdf]

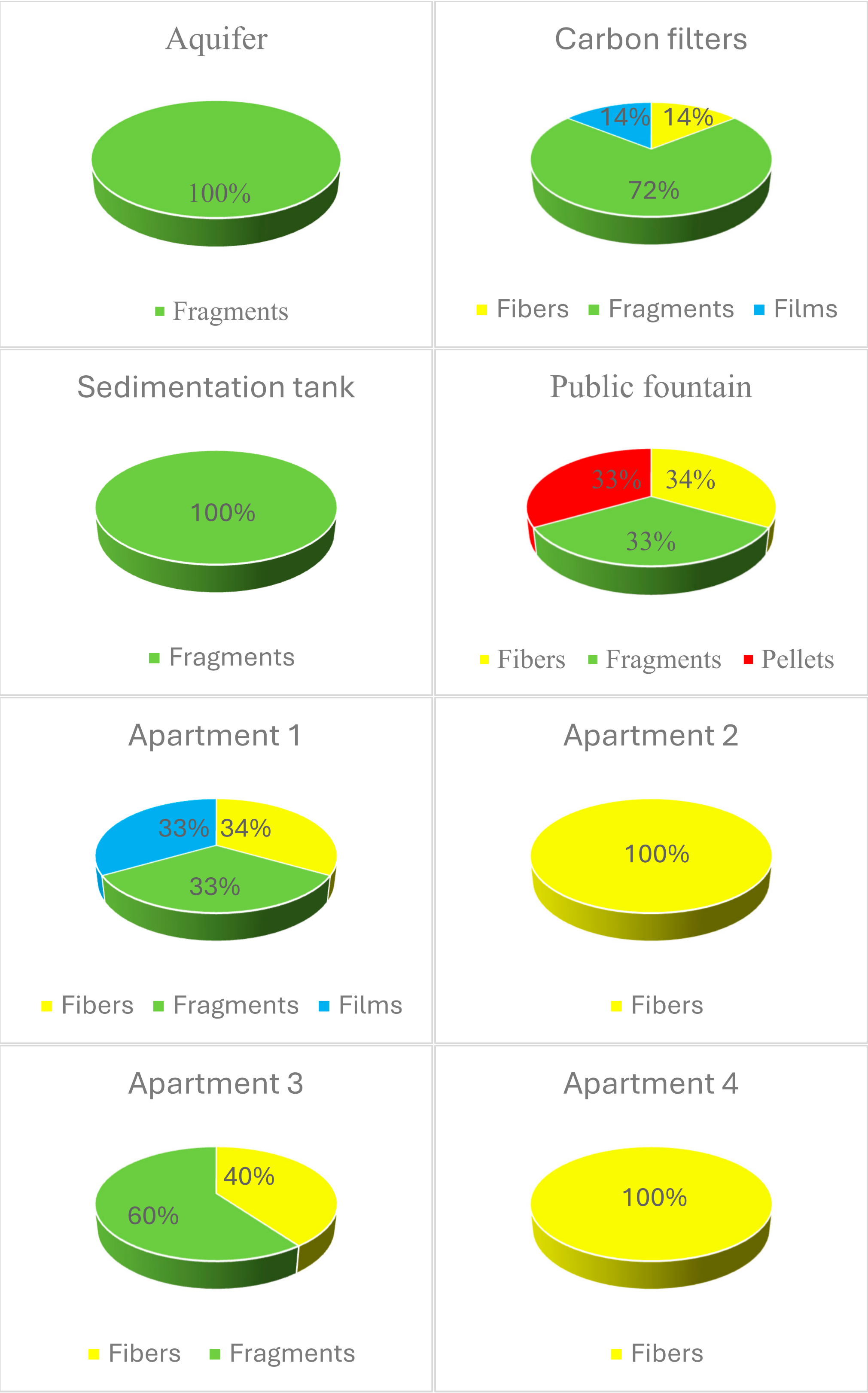
Aquifer
100%
Fragments
Carbon filters
14%
14%
72%
Fibers
Fragments
Films
Sedimentation tank
100%
Fragments
Public fountain
33%
34%
33%
Fibers
Fragments
Pellets
Apartment 1
33%
34%
33%
Fibers
Fragments
Films
Apartment 2
100%
Fibers
Apartment 3
40%
60%
Fibers
Fragments
Apartment 4
100%
Fibers

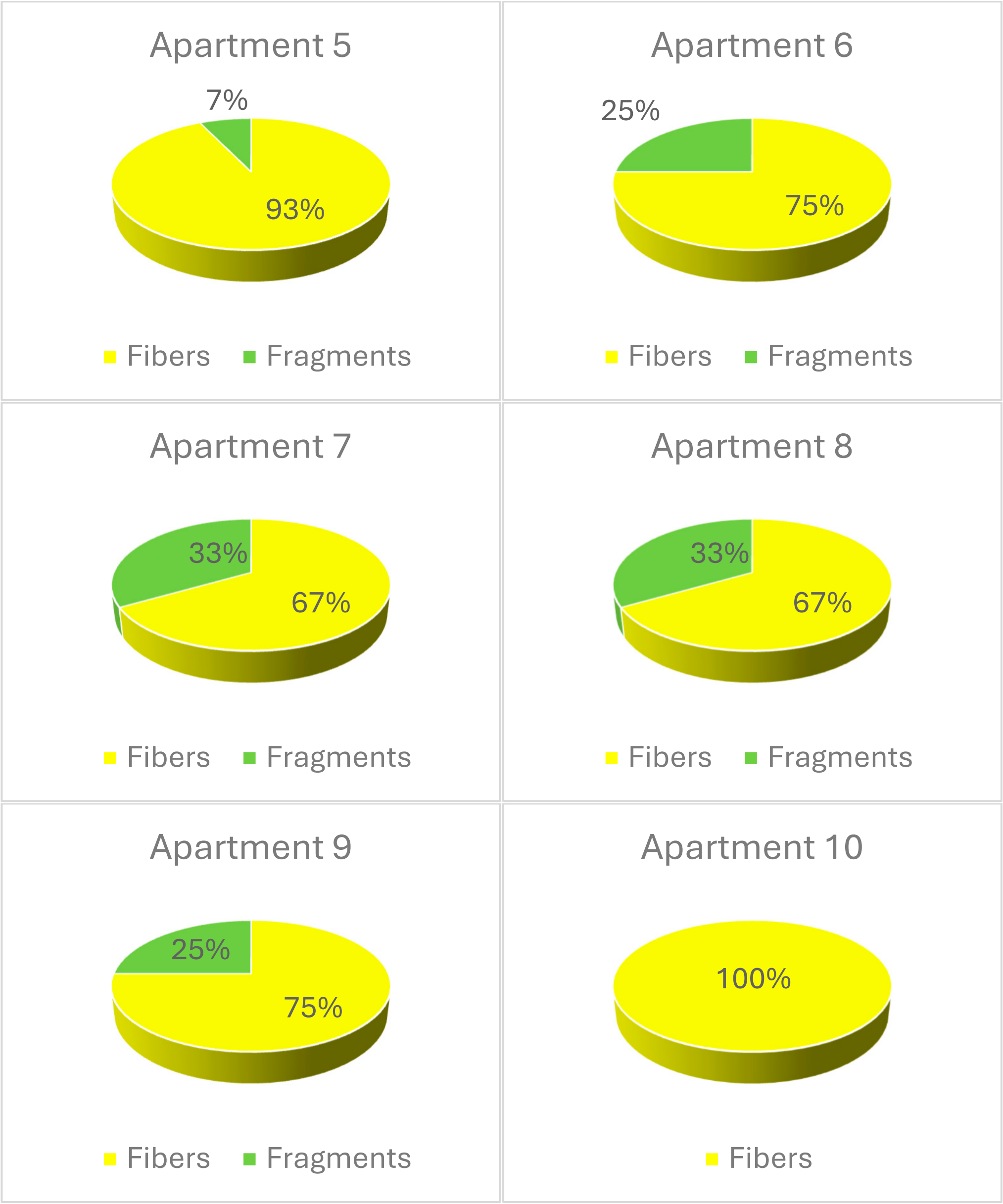

Figure S1. Shape-based classification of plastic particles detected in samples from individual sampling points.
