# Supplementary material for: From Aquifer to Tap: Comprehensive Quali-Quantitative Evaluation of Plastic Particles Along a Drinking Water Supply Chain of Milan (Northern Italy)
Source: J Xenobiot. 2026 Jan 22;16(1):18. doi: 10.3390/jox16010018 (PMC12921940; doi:10.3390/jox16010018)
Supplement: Supplementary file 1 [file jox-16-00018-s001.zip › Figure S2.pdf]

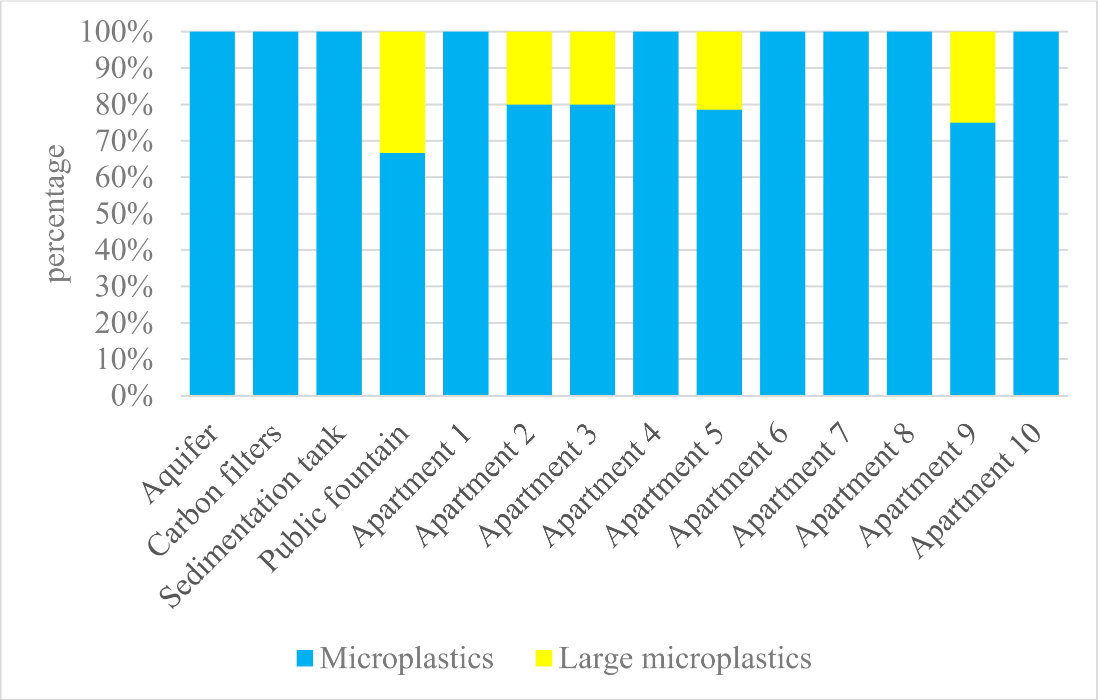

Figure S2. Size-based classification of plastic particles detected in samples from individual sampling points.
