# Supplementary material for: From Aquifer to Tap: Comprehensive Quali-Quantitative Evaluation of Plastic Particles Along a Drinking Water Supply Chain of Milan (Northern Italy)
Source: J Xenobiot. 2026 Jan 22;16(1):18. doi: 10.3390/jox16010018 (PMC12921940; doi:10.3390/jox16010018)
Supplement: Supplementary file 1 [file jox-16-00018-s001.zip › Figure S3.pdf]

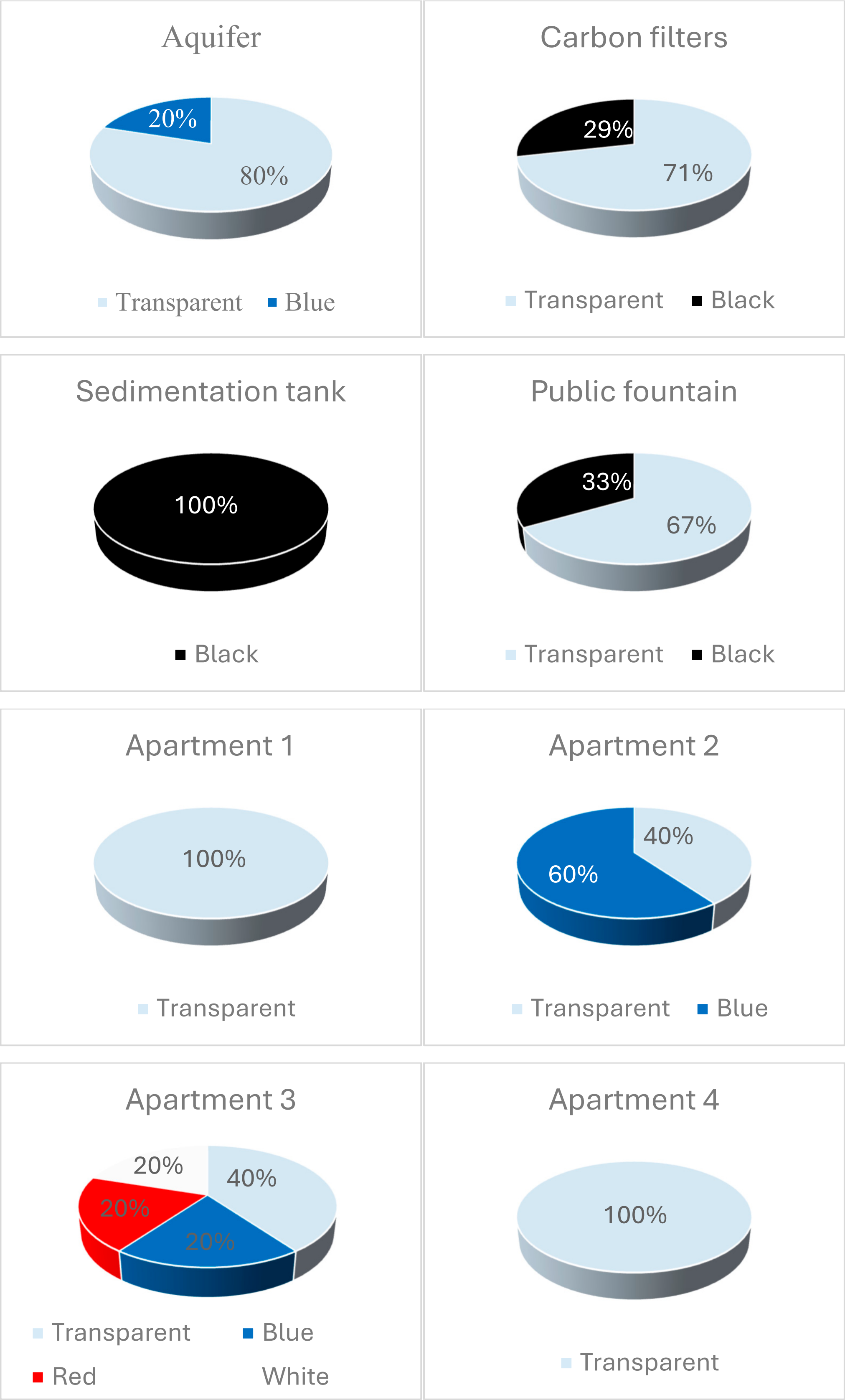
Aquifer
20%
80%
Transparent
Blue
Carbon filters
29%
71%
Transparent
Black
Sedimentation tank
100%
Black
Public fountain
33%
67%
Transparent
Black
Apartment 1
100%
Transparent
Apartment 2
40%
60%
Transparent
Blue
Apartment 3
20%
40%
20%
20%
Transparent
Blue
Red
White
Apartment 4
100%
Transparent

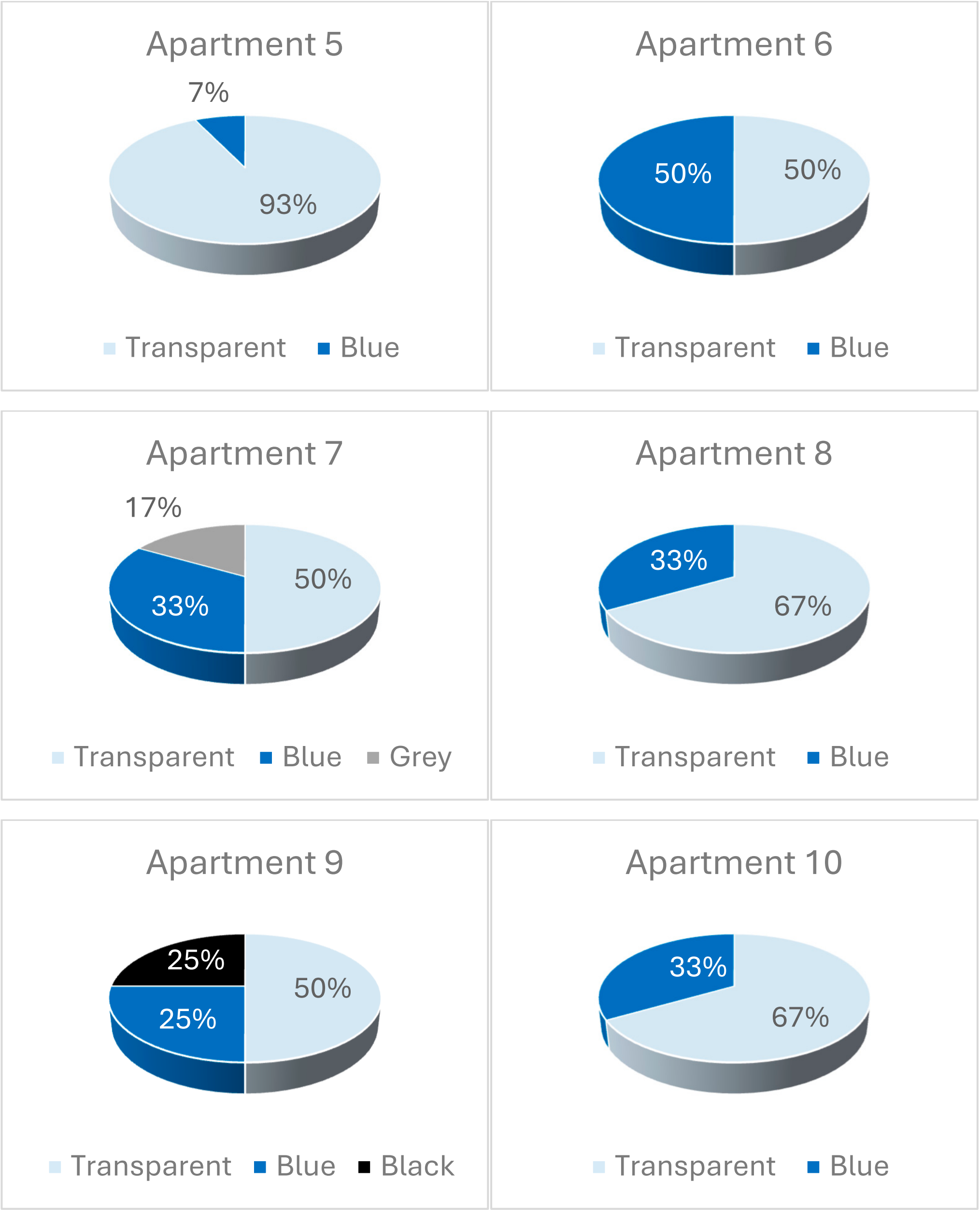

Figure S3. Color-based classification of plastic particles detected in samples from individual sampling points.
