# Supplementary material for: From Aquifer to Tap: Comprehensive Quali-Quantitative Evaluation of Plastic Particles Along a Drinking Water Supply Chain of Milan (Northern Italy)
Source: J Xenobiot. 2026 Jan 22;16(1):18. doi: 10.3390/jox16010018 (PMC12921940; doi:10.3390/jox16010018)
Supplement: Supplementary file 1 [file jox-16-00018-s001.zip › Table S1.pdf]

Table S1. Detected particles in blank samples.

Blank Filter	Code	Shape	Color	Composition	Score	TOT plastics
Aquifer, Activated carbon columns, Accumulation tank	BFFV-1	Fiber	Black	Cellulose	0.92	1
	BFFV-2	Fiber	Blue	Cellulose	0.82	
	BFFV-3	Fiber	Transparent	Cellulose	0.84	
	BFFV-4	Fiber	Transparent	Cellulose	0.83	
	BFFV-5	Fiber	Transparent	Unknown	0.94	
	BFFV-6	Fiber	Transparent	Cellulose	0.83	
	BFFV-7	Fiber	Transparent	Cellulose	0.89	
	BFFV-8	Fiber	Transparent	Polyester	0.85	
	BFFV-9	Fiber	Transparent	Cellulose	0.84	
	BFFV-10	Fiber	Transparent	Cellulose	0.80	
	BFFV-11	Fiber	Red	Cellulose	0.76	
	BFFV-12	Fiber	Transparent	Cellulose	0.85	
	BFFV-13	Fiber	Blue	Cellulose	0.89	
	BFFV-14	Fiber	Transparent	Cellulose	0.88	
	BFFV-15	Fiber	Transparent	Cellulose	0.72	
	BFFV-16	Fiber	Black	Cellulose	0.91	
	BFFV-17	Fiber	Transparent	Cellulose	0.89	
	BFFV-18	Fiber	Transparent	Cellulose	0.83	
	BFFV-19	Fragment	Transparent	Unknown	0.92	
	BFFV-20	Fiber	Blue	Cellulose	0.76	
	BFFV-21	Fiber	Transparent	Cellulose	0.78	
	BFFV-22	Fiber	Transparent	Cellulose	0.79	
	BFFV-23	Fiber	Red	Cellulose	0.75	
Public drinking fountain	BDV-1	Fiber	Transparent	Cellulose	0.90	0
	BDV-2	Fiber	Transparent	Cellulose	0.74	
	BDV-3	Fiber	Transparent	Cellulose	0.78	
	BDV-4	Fiber	Blue	Cellulose	0.78	
	BDV-5	Fiber	Red	Cellulose	0.69	
	BDV-6	Fiber	Transparent	Cellulose	0.80	
	BDV-7	Fiber	Blue	Cellulose	0.85	
	BDV-8	Fiber	Red	Cellulose	0.74	
	BDV-9	Fiber	Blue	Cellulose	0.85	
	BDV-10	Fiber	Transparent	Cellulose	0.76	
	BDV-11	Fragment	Transparent	Cellulose	0.85	
	BDV-12	Fiber	Blue	Cellulose	0.83	
	BDV-13	Fiber	Transparent	Cellulose	0.83	
	BDV-14	Fiber	Red	Cellulose	0.89	
	BDV-15	Fiber	Transparent	Cellulose	0.85	
	BDV-16	Fiber	Transparent	Cellulose	0.77	
	BDV-17	Fragment	Transparent	Cellulose	0.86	
	BDV-18	Fiber	Transparent	Cellulose	0.83	
Apartment N. 1	BCB3-1	Fiber	Transparent	Cellulose	0.77	1
	BCB3-2	Fiber	Blue	Cellulose	0.82	

	BCB3-3	Fiber	Black	Cellulose	0.70	
	BCB3-4	Fiber	Red	Cellulose	0.81	
	BCB3-5	Fiber	Transparent	Cellulose	0.84	
	BCB3-6	Film	Transparent	Polyurethane	0.97	
Apartment N. 2	BVV-1	Fiber	Blue	Cellulose	0.89	0
	BVV-2	Fiber	Transparent	Cellulose	0.82	
	BVV-3	Fiber	Transparent	Cellulose	0.88	
	BVV-4	Fiber	Transparent	Cellulose	0.75	
	BVV-5	Fiber	Transparent	Cellulose	0.70	
	BVV-6	Fiber	Black	Cellulose	0.79	
	BVV-7	Fiber	Transparent	Cellulose	0.73	
	BVV-8	Fiber	Blue	Cellulose	0.86	
	BVV-9	Fiber	Black	Cellulose	0.83	
	BVV-10	Fiber	Transparent	Cellulose	0.75	
Apartment N. 3	BPM-1	Fiber	Transparent	Cellulose	0.84	0
	BPM-2	Fiber	Transparent	Cellulose	0.84	
	BPM-3	Fiber	Blue	Cellulose	0.90	
	BPM-4	Fiber	Transparent	Cellulose	0.86	
	BPM-5	Fiber	Transparent	Cellulose	0.78	
	BPM-6	Fiber	Transparent	Cellulose	0.88	
	BPM-7	Fiber	Blue	Cellulose	0.83	
	BPM-8	Fiber	Black	Cellulose	0.76	
	BPM-9	Fragment	Transparent	Unknown	0.87	
Apartment N. 4	BVC-1	Fiber	Transparent	Polyester	0.97	2
	BVC-2	Fiber	Transparent	Polyester	0.97	
	BVC-3	Fiber	Transparent	Cellulose	0.80	
	BVC-4	Fiber	Transparent	Cellulose	0.77	
	BVC-5	Fiber	Blue	Cellulose	0.87	
	BVC-6	Fiber	Transparent	Cellulose	0.80	
	BVC-7	Fiber	Transparent	Cellulose	0.70	
	BVC-8	Fiber	Transparent	Cellulose	0.78	
	BVC-9	Fiber	Transparent	Cellulose	0.83	
	BVC-10	Fiber	Blue	Cellulose	0.91	
	BVC-11	Fiber	Transparent	Cellulose	0.85	
	BVC-12	Fiber	Transparent	Cellulose	0.79	
	BVC-13	Fiber	Blue	Cellulose	0.80	
Apartment N. 5	BVT-1	Fiber	Transparent	Cellulose	0.73	0
	BVT-2	Fiber	Transparent	Cellulose	0.84	
	BVT-3	Fiber	Transparent	Cellulose	0.75	
	BVT-4	Fiber	Transparent	Cellulose	0.86	
	BVT-5	Fiber	Transparent	Cellulose	0.74	
	BVT-6	Fiber	Transparent	Cellulose	0.81	
	BVT-7	Fiber	Transparent	Cellulose	0.83	
	BVT-8	Fiber	Transparent	Cellulose	0.73	
Apartment N. 6	BVO-1	Fiber	Black	Cellulose	0.89	0
	BVO-2	Fiber	Transparent	Cellulose	0.73	
	BVO-3	Fragment	Transparent	Unknown	0.88	

	BVO-4	Fiber	Purple	Cellulose	0.71	
	BVO-5	Fiber	Transparent	Cellulose	0.93	
	BVO-6	Fiber	Transparent	Cellulose	0.82	
	BVO-7	Fragment	Transparent	Unknown	0.93	
	BVO-8	Fiber	Purple	Cellulose	0.76	
	BVO-9	Fiber	Transparent	Cellulose	0.90	
	BVO-10	Fiber	Transparent	Cellulose	0.91	
	BVO-11	Fragment	Transparent	Zinc stearate	0.93	
	BVO-12	Fiber	Transparent	Cellulose	0.70	
	BVO-13	Fiber	Transparent	Cellulose	0.79	
	BVO-14	Fiber	Transparent	Cellulose	0.75	
	BVO-15	Fiber	Blue	Cellulose	0.88	
	BVO-16	Fiber	Transparent	Cellulose	0.81	
Apartment N. 7	BVSA-1	Fiber	Transparent	Cellulose	0.85	0
	BVSA-2	Fiber	Transparent	Cellulose	0.72	
	BVSA-3	Fiber	Transparent	Cellulose	0.93	
	BVSA-4	Fiber	Blue	Cellulose	0.93	
	BVSA-5	Fiber	Transparent	Cellulose	0.81	
	BVSA-6	Fiber	Transparent	Cellulose	0.77	
	BVSA-7	Fiber	Blue	Cellulose	0.92	
	BVSA-8	Fragment	Transparent	Unknown	0.71	
	BVSA-9	Fiber	Blue	Cellulose	0.93	
	BVSA-10	Fiber	Blue	Cellulose	0.86	
	BVSA-11	Fiber	Transparent	Cellulose	0.82	
	BVSA-12	Fiber	Transparent	Cellulose	0.83	
	BVSA-13	Fiber	Blue	Cellulose	0.82	
	BVSA-14	Fragment	Transparent	Cellulose	0.74	
	BVSA-15	Fiber	Blue	Cellulose	0.76	
	BVSA-16	Fiber	Transparent	Cellulose	0.77	
	BVSA-17	Fiber	Black	Cellulose	0.86	
	BVSA-18	Fiber	Transparent	Cellulose	0.71	
	BVSA-19	Fiber	Transparent	Cellulose	0.85	
	BVSA-20	Fiber	Transparent	Cellulose	0.75	
	BVSA-21	Fiber	Blue	Cellulose	0.88	
	BVSA-22	Fiber	Transparent	Cellulose	0.89	
	BVSA-23	Fiber	Blue	Cellulose	0.72	
	BVSA-24	Fiber	Transparent	Cellulose	0.85	
	BVSA-25	Fiber	Transparent	Cellulose	0.88	
	BVSA-26	Fiber	Transparent	Cellulose	0.94	
	BVSA-27	Fiber	Transparent	Cellulose	0.87	
Apartment N. 8	BVS-1	Fiber	Transparent	Cellulose	0.74	1
	BVS-2	Fiber	Blue	Cellulose	0.70	
	BVS-3	Fragment	White	Nitrocellulose	0.73	
	BVS-4	Fiber	Transparent	Polyester	0.89	
	BVS-5	Fiber	Transparent	Cellulose	0.75	
	BVS-6	Fiber	Black	Cellulose	0.84	
	BVS-7	Fiber	Transparent	Cellulose	0.70	

Apartment N. 9	BVVE-1	Fiber	Transparent	Cellulose	0.87	0
	BVVE-2	Fiber	Blue	Cellulose	0.80	
	BVVE-3	Fiber	Transparent	Cellulose	0.76	
	BVVE-4	Fiber	Transparent	Cellulose	0.81	
	BVVE-5	Fiber	Transparent	Cellulose	0.87	
	BVVE-6	Fiber	Transparent	Cellulose	0.84	
	BVVE-7	Fiber	Transparent	Cellulose	0.81	
	BVVE-8	Fiber	Blue	Cellulose	0.85	
	BVVE-9	Fiber	Transparent	Cellulose	0.95	
	BVVE-10	Fiber	Transparent	Cellulose	0.87	
	BVVE-11	Fiber	Blue	Cellulose	0.90	
	BVVE-12	Fragment	Transparent	Unknown	0.89	
Apartment N. 10	BVB-1	Fiber	Transparent	Cellulose	0.90	0
	BVB-2	Fiber	Transparent	Cellulose	0.83	
	BVB-3	Fiber	Transparent	Cellulose	0.83	
	BVB-4	Fiber	Transparent	Cellulose	0.83	
	BVB-5	Fiber	Transparent	Cellulose	0.80	
	BVB-6	Fiber	Transparent	Cellulose	0.83	
