# Supplementary material for: From Aquifer to Tap: Comprehensive Quali-Quantitative Evaluation of Plastic Particles Along a Drinking Water Supply Chain of Milan (Northern Italy)
Source: J Xenobiot. 2026 Jan 22;16(1):18. doi: 10.3390/jox16010018 (PMC12921940; doi:10.3390/jox16010018)
Supplement: Supplementary file 1 [file jox-16-00018-s001.zip › Table S2.pdf]

Table S2. Particles detected in the 44 samples.

AQUIFER

Filter	Code	Shape	Color	Composition	Size (mm)	Score	TOT Plastics	MP	LMP	MAP
Aquifer 1	F1-1	Fiber	Transparent	Cellulose		0.76	1	1	0	0
	F1-2	Fiber	Black	Ca carbonate		0.75				
	F1-3	Fragment	Black	Na and Al silicate		0.75				
	F1-4	Fiber	Transparent	Cellulose		0.76				
	F1-5	Fiber	Black	Cellulose		0.72				
	F1-6	Fiber	Blue	Cellulose		0.79				
	F1-7	Fiber	Blue	Cellulose		0.88				
	F1-8	Fiber	Transparent	Cellulose		0.72				
	F1-9	Fiber	Black	Cellulose		0.92				
	F1-10	Fiber	Transparent	Cellulose		0.71				
	F1-11	Fiber	Transparent	Cellulose		0.82				
	F1-12	Fiber	Transparent	Cellulose		0.83				
	F1-13	Fragment	Blue	Polyacrylate	0.11	0.73				
	F1-14	Fragment	Transparent	Unknown		0.86				
	F1-15	Fiber	Blue	Cellulose		0.74				
Aquifer 2	F2-1	Fiber	Blue	Cellulose		0.83	0	0	0	0
	F2-2	Fiber	Transparent	Cellulose		0.81				
	F2-3	Fiber	Transparent	Cellulose		0.70				

	F2-4	Fiber	Transparent	Cellulose		0.87				
	F2-5	Fiber	Black	Cellulose		0.90				
	F2-6	Fragment	Transparent	Ca stearate		0.72				
	F2-7	Fiber	Blue	Cellulose		0.76				
	F2-8	Fiber	Transparent	Cellulose		0.82				
	F2-9	Fiber	Purple	Cellulose		0.72				
	F2-10	Fiber	Purple	Cellulose		0.87				
Aquifer 3	F3-1	Fiber	Transparent	Cellulose		0.85	0	0	0	0
	F3-2	Fiber	Transparent	Cellulose		0.75				
	F3-3	Fiber	Transparent	Cellulose		0.81				
	F3-4	Fiber	Transparent	Cellulose		0.84				
	F3-5	Fiber	Blue	Cellulose		0.74				
	F3-6	Fragment	Transparent	Glass		0.73				
	F3-7	Fragment	Transparent	Glass		0.74				
	F3-8	Fiber	Transparent	Cellulose		0.73				
	F3-9	Fiber	Transparent	Cellulose		0.92				
	F3-10	Fiber	Transparent	Cellulose		0.76				
	F3-11	Fiber	Transparent	Cellulose		0.86				
	F3-12	Fragment	Transparent	Ca carbonate		0.87				
Aquifer 4	F4-1	Fiber	Transparent	Cellulose		0.82	3	3	0	0
	F4-2	Fiber	Transparent	Cellulose		0.75				
	F4-3	Fragment	Transparent	Polypropylene	0.12	0.77				
	F4-4	Fragment	Transparent	Cellulose		0.90				

	F4-5	Fiber	Transparent	Cellulose		0.77				
	F4-6	Fiber	Black	Cellulose		0.77				
	F4-7	Fiber	Transparent	Cellulose		0.83				
	F4-8	Fragment	Transparent	Quartz		0.79				
	F4-9	Fragment	Transparent	Polytetrafluoroethylene	0.17	0.84				
	F4-10	Fragment	Transparent	Polypropylene	0.21	0.97				
	F4-11	Fiber	Transparent	Cellulose		0.90				
	F4-12	Fragment	Transparent	Glass		0.70				
Aquifer 5	F5-1	Fragment	Transparent	Polypropylene	0.08	0.95	1	1	0	0
	F5-2	Fragment	Transparent	Unknown		0.88				
	F5-3	Fiber	Transparent	Ca carbonate		0.88				
	F5-4	Fragment	Transparent	Aluminium silicate		0.76				
	F5-5	Fiber	Blue	Cellulose		0.79				
	F5-6	Fragment	Transparent	Glass		0.78				
	F5-7	Fiber	Transparent	Cellulose		0.80				
	F5-8	Fragment	Transparent	Cellulose		0.73				
	F5-9	Fiber	Transparent	Cellulose		0.80				
	F5-10	Fiber	Transparent	Cellulose		0.84				
	F5-11	Fiber	Transparent	Cellulose		0.87				
	F5-12	Fragment	Transparent	Glass		0.76				

ACTIVATED CARBON COLUMNS

**Filter**	**Code**	**Shape**	**Color**	**Composition**	**Size (mm)**	**Score**	**TOT Plastics**	**MP**	**LMP**	**MAP**
From filter 1	DFI1-1	Fiber	Transparent	Cellulose		0.89	1	1	0	0
	DFI1-2	Fragment	Black	Unknown		0.74				
	DFI1-3	Fiber	Blue	Cellulose		0.74				
	DFI1-4	Fiber	Transparent	Cellulose		0.81				
	DFI1-5	Fiber	Black	Cellulose		0.75				
	DFI1-6	Fragment	Transparent	Cellulose		0.81				
	DFI1-7	Fragment	Transparent	Unknown		0.86				
	DFI1-8	Film	Transparent	Polypropylene	0.31	0.84				
	DFI1-9	Fragment	Transparent	Cellulose		0.88				
	DFI1-10	Fiber	Transparent	Cellulose		0.90				
	DFI1-11	Fiber	Transparent	Cellulose		0.88				
From filter 2	DFI2-1	Fiber	Blue	Cellulose		0.76	6	6	0	0
	DFI2-2	Fragment	Transparent	Polytetrafluoroethylene	0.15	0.93				
	DFI2-3	Fragment	Transparent	Polytetrafluoroethylene	0.27	0.88				

	DFI2-4	Fragment	Transparent	Unknown		0.93				
	DFI2-5	Fiber	Transparent	Unknown		0.80				
	DFI2-6	Fiber	Transparent	Cellulose		0.76				
	DFI2-7	Fiber	Transparent	Cellulose		0.90				
	DFI2-8	Fiber	Transparent	Cellulose		0.72				
	DFI2-9	Fiber	Transparent	Cellulose		0.70				
	DFI2-10	Fragment	Black	Polytetrafluoroethylene	0.08	0.95				
	DFI2-11	Fiber	Transparent	Cellulose		0.84				
	DFI2-12	Fragment	Black	Polytetrafluoroethylene	0.18	0.77				
	DFI2-13	Fiber	Transparent	Cellulose		0.92				
	DFI2-14	Fiber	Transparent	Cellulose		0.70				
	DFI2-15	Fiber	Transparent	Polyamide	0.82	0.93				
	DFI2-16	Fiber	Transparent	Unknown		0.82				
	DFI2-17	Fiber	Blue	Cellulose		0.81				
	DFI2-18	Fiber	Black	Cellulose		0.90				
	DFI2-19	Fragment	Transparent	Cellulose		0.84				

	DFI2-20	Fragment	Transparent	Polytetrafluoroethylene	0.12	0.90				
	DFI2-21	Fiber	Transparent	Cellulose		0.93				
	DFI2-22	Fiber	Blue	Cellulose		0.75				
	DFI2-23	Fiber	Black	Cellulose		0.90				
	DFI2-24	Fiber	Transparent	Cellulose		0.82				
From filter3	DFI3-1	Fiber	Transparent	Cellulose		0.80	0	0	0	0
	DFI3-2	Fiber	Transparent	Cellulose		0.76				
	DFI3-3	Fiber	Transparent	Cellulose		0.82				
	DFI3-4	Fiber	Transparent	Cellulose		0.73				
	DFI3-5	Fiber	Transparent	Cellulose		0.92				
	DFI3-6	Fiber	Transparent	Cellulose		0.87				
	DFI3-7	Fiber	Black	Cellulose		0.86				
	DFI3-8	Fragment	Transparent	Cellulose		0.81				
	DFI3-9	Fragment	Transparent	Cellulose		0.87				
	DFI3-10	Fiber	Black	Cellulose		0.81				
	DFI3-11	Fiber	Transparent	Cellulose		0.86				

	DFI3-12	Fiber	Transparent	Cellulose		0.70				
	DFI3-13	Fragment	Transparent	Unknown		0.89				

ACCUMULATION TANK

Filter	Code	Shape	Color	Composition	Size (mm)	Score	TOT Plastics	MP	LMP	MAP
After tank 1	DVA1-1	Fiber	Transparent	Cellulose		0.87	1	1	0	0
	DVA1-2	Fragment	Transparent	Cellulose		0.87				
	DVA1-3	Fragment	Transparent	Aluminium silicate		0.82				
	DVA1-4	Fiber	Transparent	Cellulose		0.83				
	DVA1-5	Fiber	Transparent	Cellulose		0.77				
	DVA1-6	Fragment	Black	Polyacrylate	0.03	0.70				
	DVA1-7	Fiber	Transparent	Cellulose		0.75				
	DVA1-8	Fiber	Transparent	Cellulose		0.91				
	DVA1-9	Fiber	Blue	Cellulose		0.85				
	DVA1-10	Fiber	Transparent	Cellulose		0.83				
	DVA1-11	Fiber	Transparent	Cellulose		0.84				
	DVA1-12	Fiber	Transparent	Cellulose		0.73				

	DVA1-13	Fiber	Transparent	Cellulose		0.87				
After tank 2	DVA2-1	Fragment	Transparent	Unknown		0.76	0	0	0	0
	DVA2-2	Fiber	Transparent	Cellulose		0.93				
	DVA2-3	Fiber	Transparent	Cellulose		0.71				
	DVA2-4	Fiber	Blue	Cellulose		0.79				
	DVA2-5	Fiber	Blue	Cellulose		0.70				
	DVA2-6	Fiber	Transparent	Unknown		0.86				
	DVA2-7	Fiber	Transparent	Cellulose		0.94				
	DVA2-8	Fragment	Transparent	Glass		0.73				
	DVA2-9	Fragment	Transparent	Glass		0.76				
	DVA2-10	Fiber	Blue	Cellulose		0.85				
	DVA2-11	Fragment	Yellow	Na and Al silicate		0.83				
	DVA2-12	Fiber	Transparent	Cellulose		0.89				
	DVA2-13	Fragment	Transparent	Na and Al silicate		0.84				
	DVA2-14	Fiber	Transparent	Cellulose		0.80				

After tank 3	DVA3-1	Fiber	Transparent	Cellulose		0.78	0	0	0	0
	DVA3-2	Fiber	Transparent	Cellulose		0.87				
	DVA3-4	Fragment	Transparent	Cellulose		0.92				
	DVA3-5	Fiber	Transparent	Cellulose		0.78				
	DVA3-6	Fiber	Transparent	Cellulose		0.92				
	DVA3-7	Fiber	Transparent	Cellulose		0.74				
	DVA3-8	Fiber	Transparent	Cellulose		0.78				
	DVA3-9	Fiber	Transparent	Unknown		0.89				
	DVA3-10	Fiber	Transparent	Cellulose		0.89				
	DVA3-11	Fiber	Black	Cellulose		0.84				
	DVA3-12	Fiber	Transparent	Cellulose		0.87				
	DVA3-13	Fiber	Blue	Cellulose		0.74				
	DVA3-14	Fragment	Black	Talcum		0.88				
	DVA3-15	Fiber	Transparent	Cellulose		0.85				
	DVA3-16	Fiber	Black	Cellulose		0.86				

PUBLIC DRINKING FOUNTAIN

Filter	Code	Shape	Color	Composition	Size (mm)	Score	TOT Plastics	MP	LMP	MAP
Fountain 1	DV1-1	Fiber	Transparent	Cellulose		0.89	1	0	1	0
	DV1-2	Fiber	Black	Cellulose		0.70				
	DV1-3	Fiber	Transparent	Cellulose		0.69				
	DV1-4	Fiber	Black	Cellulose		0.91				
	DV1-5	Fiber	Transparent	Cellulose		0.70				
	DV1-6	Fiber	Transparent	Cellulose		0.80				
	DV1-7	Fiber	Blue	Cellulose		0.88				
	DV1-8	Fiber	Transparent	Cellulose		0.79				
	DV1-9	Fragment	Transparent	Quartz		0.65				
	DV1-10	Fiber	Transparent	Cellulose		0.84				
	DV1-11	Fiber	Black	Polyester	3.60	0.96				
	DV1-12	Fiber	Transparent	Cellulose		0.79				
	DV1-13	Fiber	Transparent	Cellulose		0.81				
	DV1-14	Fiber	Transparent	Cellulose		0.70				
	DV1-15	Fiber	Transparent	Cellulose		0.90				

	DV1-16	Fiber	Blue	Cellulose		0.83				
	DV1-17	Fiber	Blue	Cellulose		0.81				
	DV1-18	Fiber	Transparent	Cellulose		0.70				
Fountain 2	DV2-1	Fragment	Grey	Aluminium sulphate		0.66	1	1	0	0
	DV2-2	Fiber	Transparent	Cellulose		0.86				
	DV2-3	Fiber	Transparent	Cellulose		0.73				
	DV2-4	Fiber	Transparent	Cellulose		0.79				
	DV2-5	Fiber	Blue	Cellulose		0.71				
	DV2-6	Fiber	Transparent	Cellulose		0.74				
	DV2-7	Fiber	Black	Unknown		0.91				
	DV2-8	Fiber	Transparent	Cellulose		0.85				
	DV2-9	Fiber	Transparent	Cellulose		0.90				
	DV2-10	Fiber	Blue	Cellulose		0.85				
	DV2-11	Fiber	Blue	Cellulose		0.77				
	DV2-12	Fiber	Transparent	Cellulose		0.80				
	DV2-13	Fiber	Transparent	Cellulose		0.76				

	DV2-14	Fiber	Blue	Cellulose		0.90				
	DV2-15	Fiber	Transparent	Unknown		0.87				
	DV2-16	Fragment	Grey	Aluminium sulphate		0.68				
	DV2-17	Fiber	Transparent	Cellulose		0.89				
	DV2-18	Fiber	Transparent	Cellulose		0.77				
	DV2-19	Fragment	Grey	Aluminium sulphate		0.60				
	DV2-20	Fragment	Transparent	Polyurethane	0.13	0.95				
	DV2-21	Fiber	Transparent	Cellulose		0.73				
	DV2-22	Fragment	Grey	Aluminium sulphate		0.68				
	DV2-23	Fragment	Grey	Aluminium sulphate		0.61				
Fountain 3	DV3-1	Fiber	Blue	Unknown		0.93	1	1	0	0
	DV3-2	Fiber	Transparent	Cellulose		0.80				
	DV3-3	Fiber	Red	Cellulose		0.87				
	DV3-4	Fiber	Blue	Cellulose		0.73				
	DV3-5	Fiber	Transparent	Cellulose		0.92				
	DV3-6	Fiber	Blue	Cellulose		0.90				

	DV3-7	Fiber	Transparent	Cellulose		0.84				
	DV3-8	Fiber	Transparent	Cellulose		0.85				
	DV3-9	Fiber	Transparent	Cellulose		0.80				
	DV3-10	Fiber	Transparent	Cellulose		0.81				
	DV3-11	Fiber	Transparent	Unknown		0.93				
	DV3-12	Fragment	Transparent	Ca stearate		0.73				
	DV3-13	Fiber	Transparent	Cellulose		0.85				
	DV3-14	Fiber	Blue	Unknown		0.87				
	DV3-15	Fragment	Black	Silicone		0.70				
	DV3-16	Fragment	Transparent	Ca stearate		0.79				
	DV3-17	Pellet	Transparent	Polyurethane	0.10	0.96				
	DV3-18	Fiber	Blue	Cellulose		0.85				
	DV3-19	Fiber	Blue	Cellulose		0.80				
	DV3-20	Fiber	Transparent	Cellulose		0.82				

APARTMENT N. 1

Filter	Code	Shape	Color	Composition	Size (mm)	Score	TOT Plastics	MP	LMP	MAP
Tap of apartment 1.1	CB1-1	Fiber	Blue	Cellulose		0.76	1	1	0	0
	CB1-2	Fiber	Blue	Cellulose		0.71				
	CB1-3	Fiber	Transparent	Cellulose		0.65				
	CB1-4	Fiber	Transparent	Cellulose		0.66				
	CB1-5	Fiber	Transparent	Cellulose		0.94				
	CB1-6	Fiber	Transparent	Cellulose		0.69				
	CB1-7	Fragment	Transparent	Polyurethane	0.21	0.81				
	CB1-8	Fiber	Transparent	Cellulose		0.82				
	CB1-9	Fiber	Transparent	Cellulose		0.82				
	CB1-10	Fiber	Transparent	Cellulose		0.92				
	CB1-11	Fiber	Transparent	Cellulose		0.82				
	CB1-12	Fiber	Transparent	Cellulose		0.70				
	CB1-13	Fiber	Transparent	Cellulose		0.68				
	CB1-14	Fiber	Blue	Cellulose		0.85				

	CB1-15	Fiber	Transparent	Cellulose		0.72				
	CB1-16	Fiber	Transparent	Cellulose		0.91				
	CB1-17	Fragment	Transparent	Ca stearate		0.75				
Tap of apartment 1.2	CB2-1	Fiber	Transparent	Cellulose		0.83	0	0	0	0
	CB2-2	Fiber	Blue	Cellulose		0.72				
	CB2-3	Fragment	Transparent	Ca stearate		0.79				
	CB2-4	Fiber	Transparent	Cellulose		0.94				
	CB2-5	Fiber	Black	Cellulose		0.76				
	CB2-6	Fragment	Transparent	Cellulose		0.85				
	CB2-7	Fiber	Red	Cellulose		0.78				
	CB2-8	Fragment	Transparent	Aragonite		0.73				
	CB2-9	Fiber	Red	Cellulose		0.75				
	CB2-10	Fiber	Blue	Cellulose		0.78				
	CB2-11	Fiber	Transparent	Cellulose		0.85				
	CB2-12	Fiber	Transparent	Cellulose		0.66				
	CB2-13	Fiber	Transparent	Cellulose		0.82				

	CB2-14	Fiber	Transparent	Cellulose		0.80				
	CB2-15	Fiber	Black	Cellulose		0.77				
	CB2-16	Fiber	Black	Cellulose		0.79				
	CB2-17	Fiber	Black	Cellulose		0.79				
	CB2-18	Fiber	Blue	Cellulose		0.78				
	CB2-19	Fiber	Transparent	Cellulose		0.95				
	CB2-20	Fiber	Transparent	Cellulose		0.77				
	CB2-21	Fiber	Transparent	Cellulose		0.77				
	CB2-22	Fiber	Transparent	Cellulose		0.81				
	CB2-23	Fiber	Transparent	Cellulose		0.87				
	CB2-24	Fiber	Transparent	Cellulose		0.75				
	CB2-25	Fiber	Transparent	Cellulose		0.65				
	CB2-26	Fiber	Transparent	Cellulose		0.89				
	CB2-27	Fiber	Transparent	Cellulose		0.86				
	CB2-28	Fiber	Transparent	Cellulose		0.84				
Tap of apartment 1.3	CB3-1	Fragment	Grey	Quartz		0.92	2	2	0	0

	CB3-2	Fiber	Transparent	Cellulose		0.81					
	CB3-3	Fiber	Transparent	Polyacrylate	0.23	0.74					
	CB3-4	Fragment	Black	Aluminium sulphate		0.71					
	CB3-5	Fragment	Black	Aluminium sulphate		0.74					
	CB3-6	Fragment	Black	Aluminium sulphate		0.79					
	CB3-7	Fiber	Transparent	Cellulose		0.76					
	CB3-8	Fiber	Transparent	Cellulose		0.75					
	CB3-9	Fiber	Transparent	Cellulose		0.83					
	CB3-10	Fragment	Black	Aluminium sulphate		0.64					
	CB3-11	Fiber	Transparent	Unknown		0.75					
	CB3-12	Fiber	Transparent	Cellulose		0.73					
	CB3-13	Fragment	Black	Aluminium sulphate		0.65					
	CB3-14	Fragment	Transparent	Cellulose		0.78					
	CB3-15	Fiber	Transparent	Cellulose		0.91					
	CB3-16	Fiber	Transparent	Cellulose		0.87					
	CB3-17	Fiber	Transparent	Cellulose		0.91					

	CB3-18	Fiber	Transparent	Cellulose		0.94				
	CB3-19	Fiber	Transparent	Cellulose		0.85				
	CB3-20	Film	Transparent	Polystyrene	0.19	0.96				
	CB3-21	Fiber	Transparent	Cellulose		0.75				
	CB3-22	Fiber	Transparent	Cellulose		0.79				
	CB3-23	Fragment	Black	Aluminium sulphate		0.57				

APARTMENT N. 2

Filter	Code	Shape	Color	Composition	Size (mm)	Score	TOT Plastics	MP	LMP	MAP
Tap of apartment 2.1	VV1-1	Fiber	Transparent	Cellulose		0.84	2	2	0	0
	VV1-2	Fiber	Blue	Cellulose		0.80				
	VV1-3	Fragment	Grey	Aluminium sulphate		0.60				
	VV1-4	Fiber	Transparent	Cellulose		0.72				
	VV1-5	Fiber	Black	Cellulose		0.80				
	VV1-6	Fragment	Grey	Aluminium sulphate		0.62				
	VV1-7	Fiber	Blue	Cellulose		0.74				
	VV1-8	Fiber	Transparent	Cellulose		0.76				

	VV1-9	Fiber	Transparent	Cellulose		0.69				
	VV1-10	Fiber	Transparent	Cellulose		0.70				
	VV1-11	Fiber	Blue	Polyacrylate	0.60	0.70				
	VV1-12	Fiber	Red	Cellulose		0.83				
	VV1-13	Fiber	Transparent	Cellulose		0.83				
	VV1-14	Fiber	Transparent	Polyester	0.16	0.91				
	VV1-15	Fiber	Transparent	Cellulose		0.77				
Tap of apartment 2.2	VV2-1	Fragment	Transparent	Unknown		0.93	1	1	0	0
	VV2-2	Fiber	Transparent	Cellulose		0.83				
	VV2-3	Fiber	Transparent	Cellulose		0.78				
	VV2-4	Fiber	Transparent	Cellulose		0.69				
	VV2-5	Fiber	Transparent	Cellulose		0.88				
	VV2-6	Fiber	Blue	Cellulose		0.77				
	VV2-7	Fragment	Transparent	Ca stearate		0.79				
	VV2-8	Fiber	Transparent	Cellulose		0.75				
	VV2-9	Fiber	Transparent	Cellulose		0.69				

	VV2-10	Fiber	Transparent	Cellulose		0.81				
	VV2-11	Fiber	Transparent	Cellulose		0.86				
	VV2-12	Fiber	Transparent	Cellulose		0.94				
	VV2-13	Fiber	Transparent	Cellulose		0.76				
	VV2-14	Fiber	Blue	Polyester	0.38	0.94				
	VV2-15	Fiber	Transparent	Cellulose		0.90				
	VV2-16	Fiber	Transparent	Cellulose		0.74				
Tap of apartment 2.3	VV3-1	Fiber	Blue	Cellulose		0.74	2	1	1	0
	VV3-2	Fiber	Transparent	Cellulose		0.75				
	VV3-3	Fiber	Transparent	Cellulose		0.82				
	VV3-4	Fiber	Transparent	Cellulose		0.83				
	VV3-5	Fiber	Transparent	Polyester	0.58	0.96				
	VV3-6	Fiber	Transparent	Cellulose		0.74				
	VV3-7	Fiber	Black	Cellulose		0.72				
	VV3-8	Fiber	Transparent	Cellulose		0.72				
	VV3-9	Fiber	Transparent	Cellulose		0.74				

	VV3-10	Fragment	Transparent	Unknown		0.70				
	VV3-11	Fiber	Blue	Polyester	2.76	0.97				
	VV3-12	Fragment	Transparent	Na and Al silicate		0.84				

APARTMENT N. 3

Filter	Code	Shape	Color	Composition	Size (mm)	Score	TOT Plastics	MP	LMP	MAP
Tap of apartment 3.1	PM1-1	Fragment	Transparent	Unknown		0.93	0	0	0	0
	PM1-2	Fragment	Transparent	Ca stearate		0.71				
	PM1-3	Fragment	Transparent	Unknown		0.83				
	PM1-4	Fiber	Transparent	Cellulose		0.74				
	PM1-5	Fiber	Transparent	Cellulose		0.76				
	PM1-6	Fiber	Transparent	Cellulose		0.70				
	PM1-7	Fragment	Transparent	Cellulose		0.84				
	PM1-8	Fragment	Transparent	Unknown		0.84				
	PM1-9	Fragment	Transparent	Ca stearate		0.87				
	PM1-10	Fiber	Transparent	Cellulose		0.78				

	PM1-11	Fiber	Transparent	Cellulose		0.87				
	PM1-12	Fragment	Transparent	Unknown		0.86				
	PM1-13	Fiber	Transparent	Cellulose		0.81				
	PM1-14	Fiber	Transparent	Cellulose		0.70				
	PM1-15	Fragment	Black	Ca stearate		0.73				
	PM1-16	Fiber	Transparent	Cellulose		0.83				
	PM1-17	Fiber	Transparent	Cellulose		0.80				
	PM1-18	Fiber	Blue	Cellulose		0.71				
	PM1-19	Fragment	Transparent	Unknown		0.78				
	PM1-20	Fragment	Transparent	Ca stearate		0.79				
Tap of apartment 3.2	PM2-1	Fiber	Transparent	Cellulose		0.78	3	3	0	0
	PM2-2	Fiber	Transparent	Polyester	0.83	0.90				
	PM2-3	Fiber	Transparent	Cellulose		0.76				
	PM2-4	Fiber	Transparent	Cellulose		0.88				
	PM2-5	Fragment	Transparent	Ca stearate		0.72				
	PM2-6	Fiber	Red	Cellulose		0.86				

	PM2-7	Fiber	Transparent	Cellulose		0.93				
	PM2-8	Fiber	Blue	Cellulose		0.65				
	PM2-9	Fragment	White	Unknown		0.90				
	PM2-10	Fiber	Transparent	Cellulose		0.76				
	PM2-11	Fiber	Transparent	Cellulose		0.85				
	PM2-12	Fiber	Transparent	Cellulose		0.78				
	PM2-13	Fragment	Blue	Polyacrylate	0.19	0.77				
	PM2-14	Fiber	Transparent	Cellulose		0.79				
	PM2-15	Fiber	Transparent	Cellulose		0.72				
	PM2-16	Fiber	Transparent	Cellulose		0.75				
	PM2-17	Fiber	Transparent	Unknown		0.81				
	PM2-18	Fragment	White	Unknown		0.93				
	PM2-19	Fiber	Transparent	Cellulose		0.82				
	PM2-20	Fiber	Black	Unknown		0.94				
	PM2-21	Fiber	Blue	Cellulose		0.78				
	PM2-22	Fragment	Transparent	Polyurethane	0.25	0.81				

	PM2-23	Fiber	Transparent	Cellulose		0.78				
	PM2-24	Fiber	Red	Cellulose		0.75				
	PM2-25	Fiber	Black	Cellulose		0.80				
	PM2-26	Fiber	Transparent	Cellulose		0.78				
Tap of apartment 3.3	PM3-1	Fragment	Black	Unknown		0.87	2	1	1	0
	PM3-2	Fragment	Grey	Aluminium sulphate		0.65				
	PM3-3	Fiber	Transparent	Cellulose		0.77				
	PM3-4	Fiber	Blue	Cellulose		0.76				
	PM3-5	Fiber	Transparent	Cellulose		0.87				
	PM3-6	Fiber	Black	Cellulose		0.95				
	PM3-7	Fiber	Blue	Cellulose		0.84				
	PM3-8	Fiber	Transparent	Cellulose		0.86				
	PM3-9	Fiber	Transparent	Cellulose		0.84				
	PM3-10	Fiber	Transparent	Cellulose		0.86				
	PM3-11	Fiber	Transparent	Cellulose		0.68				
	PM3-12	Fiber	Transparent	Cellulose		0.89				

	PM3-13	Fiber	Transparent	Cellulose		0.76				
	PM3-14	Fiber	Transparent	Cellulose		0.88				
	PM3-15	Fiber	Transparent	Cellulose		0.71				
	PM3-16	Fiber	Transparent	Cellulose		0.84				
	PM3-17	Fiber	Transparent	Cellulose		0.95				
	PM3-18	Fiber	Transparent	Cellulose		0.74				
	PM3-19	Fiber	Transparent	Cellulose		0.78				
	PM3-20	Fiber	Transparent	Unknown		0.96				
	PM3-21	Fiber	Transparent	Cellulose		0.73				
	PM3-22	Fiber	Red	Polyester	1.78	0.94				
	PM3-23	Fiber	Transparent	Cellulose		0.81				
	PM3-24	Fiber	Transparent	Cellulose		0.87				
	PM3-25	Fiber	Blue	Cellulose		0.79				
	PM3-26	Fiber	Blue	Cellulose		0.77				
	PM3-27	Fiber	Blue	Cellulose		0.82				
	PM3-28	Fiber	Transparent	Cellulose		0.90				

	PM3-29	Fiber	Transparent	Cellulose		0.90				
	PM3-30	Fragment	White	Acrylonitrile-butadiene-styrene	0.24	0.84				
	PM3-31	Fiber	Transparent	Cellulose		0.79				
	PM3-32	Fiber	Transparent	Cellulose		0.73				
	PM3-33	Fiber	Transparent	Cellulose		0.81				
	PM3-34	Fiber	Black	Cellulose		0.90				

APARTMENT N. 4

**Filter**	**Code**	**Shape**	**Color**	**Composition**	**Size (mm)**	**Score**	**TOT Plastics**	**MP**	**LMP**	**MAP**
Tap of apartment 4.1	VC1-1	Fiber	Transparent	Polyester	0.20	0.88	3	3	0	0
	VC1-2	Fiber	Transparent	Cellulose		0.81				
	VC1-3	Fiber	Transparent	Cellulose		0.78				
	VC1-4	Fiber	Black	Cellulose		0.83				
	VC1-5	Fiber	Transparent	Polyester	0.45	0.97				
	VC1-7	Fiber	Blue	Cellulose		0.73				
	VC1-9	Fiber	Transparent	Polyester	0.18	0.97				

	VC1-10	Fragment	Transparent	Ca stearate		0.71				
	VC1-11	Fiber	Transparent	Cellulose		0.93				
	VC1-12	Fiber	Transparent	Cellulose		0.89				
	VC1-13	Fragment	Grey	Aluminium sulphate		0.70				
Tap of apartment 4.2	VC2-1	Fiber	Transparent	Polyester	0.75	0.96	2	2	0	0
	VC2-2	Fiber	Transparent	Cellulose		0.77				
	VC2-3	Fiber	Transparent	Cellulose		0.70				
	VC2-4	Fiber	Transparent	Cellulose		0.91				
	VC2-5	Fiber	Transparent	Cellulose		0.89				
	VC2-6	Fiber	Transparent	Polyester-Polyamide	0.90	0.98				
	VC2-7	Fiber	Transparent	Cellulose		0.84				
	VC2-8	Fiber	Transparent	Cellulose		0.81				
	VC2-9	Fiber	Transparent	Cellulose		0.76				
	VC2-10	Fiber	Transparent	Cellulose		0.85				
	VC2-11	Fiber	Transparent	Cellulose		0.87				
	VC2-12	Fiber	Transparent	Cellulose		0.85				

	VC2-13	Fragment	Transparent	Unknown		0.89				
Tap of apartment 4.3	VC3-1	Fiber	Blue	Cellulose		0.84	2	2	0	0
	VC3-2	Fiber	Transparent	Polyester	0.14	0.98				
	VC3-3	Fiber	Transparent	Cellulose		0.91				
	VC3-4	Fiber	Transparent	Cellulose		0.77				
	VC3-5	Fiber	Transparent	Polyester	0.25	0.96				
	VC3-6	Fiber	Transparent	Cellulose		0.89				
	VC3-7	Fiber	Transparent	Cellulose		0.84				
	VC3-8	Fiber	Transparent	Cellulose		0.85				
	VC3-9	Fiber	Transparent	Cellulose		0.86				
	VC3-10	Fiber	Transparent	Cellulose		0.76				
	VC3-11	Fiber	Blue	Unknown		0.93				
	VC3-12	Fiber	Transparent	Cellulose		0.71				

APARTMENT N. 5

Filter	Code	Shape	Color	Composition	Size (mm)	Score	TOT Plastics	MP	LMP	MAP
Tap of apartment 5.1	VT1-1	Fragment	Transparent	Arabic gum		0.83	3	2	1	0
	VT1-2	Fiber	Blue	Cellulose		0.85				
	VT1-3	Fiber	Transparent	Cellulose		0.77				
	VT1-4	Fiber	Transparent	Cellulose		0.93				
	VT1-5	Fiber	Transparent	Cellulose		0.79				
	VT1-6	Fiber	Transparent	Cellulose		0.86				
	VT1-7	Fiber	Transparent	Cellulose		0.71				
	VT1-8	Fragment	Blue	Polyacrylate	0.08	0.77				
	VT1-9	Fiber	Blue	Cellulose		0.75				
	VT1-10	Fiber	Transparent	Cellulose		0.70				
	VT1-11	Fiber	Transparent	Polyester	0.33	0.97				
	VT1-12	Fiber	Transparent	Cellulose		0.78				
	VT1-13	Fiber	Transparent	Cellulose		0.70				
	VT1-14	Fiber	Transparent	Cellulose		0.75				

	VT1-15	Fragment	Transparent	Glass		0.84				
	VT1-16	Fiber	Transparent	Cellulose		0.81				
	VT1-17	Fiber	Transparent	Cellulose		0.80				
	VT1-18	Fiber	Transparent	Cellulose		0.90				
	VT1-19	Fiber	Transparent	Cellulose		0.75				
	VT1-20	Fiber	Transparent	Polyester	1.17	0.93				
	VT1-21	Fiber	Black	Cellulose		0.81				
Tap of apartment 5.2	VT2-1	Fiber	Transparent	Polyester	0.40	0.74	5	3	2	0
	VT2-2	Fiber	Transparent	Cellulose		0.78				
	VT2-3	Fiber	Transparent	Cellulose		0.81				
	VT2-4	Fiber	Transparent	Cellulose		0.79				
	VT2-5	Fiber	Transparent	Cellulose		0.85				
	VT2-6	Fiber	Transparent	Cellulose		0.73				
	VT2-7	Fiber	Transparent	Cellulose		0.77				
	VT2-8	Fiber	Transparent	Cellulose		0.81				
	VT2-9	Fiber	Transparent	Cellulose		0.83				

	VT2-10	Fiber	Transparent	Cellulose		0.93				
	VT2-11	Fiber	Transparent	Cellulose		0.86				
	VT2-12	Fiber	Blue	Cellulose		0.76				
	VT2-13	Fiber	Transparent	Cellulose		0.85				
	VT2-14	Fiber	Transparent	Polyester	1.10	0.71				
	VT2-15	Fiber	Transparent	Cellulose		0.86				
	VT2-16	Fiber	Transparent	Cellulose		0.81				
	VT2-17	Fiber	Transparent	Cellulose		0.86				
	VT2-18	Fiber	Transparent	Cellulose		0.93				
	VT2-19	Fiber	Blue	Cellulose		0.82				
	VT2-20	Fiber	Blue	Cellulose		0.82				
	VT2-21	Fiber	Transparent	Polyester	1.06	0.95				
	VT2-22	Fiber	Transparent	Cellulose		0.91				
	VT2-23	Fiber	Blue	Cellulose		0.87				
	VT2-24	Fiber	Transparent	Cellulose		0.84				
	VT2-25	Fiber	Transparent	Cellulose		0.87				

	VT2-26	Fiber	Transparent	Polyester	0.98	0.94				
	VT2-27	Fiber	Transparent	Polyester	0.95	0.96				
	VT2-28	Fiber	Transparent	Cellulose		0.70				
	VT2-29	Fiber	Transparent	Cellulose		0.81				
	VT2-30	Fiber	Transparent	Cellulose		0.92				
	VT2-31	Fiber	Transparent	Cellulose		0.76				
	VT2-32	Fiber	Transparent	Cellulose		0.71				
	VT2-33	Fiber	Transparent	Cellulose		0.80				
Tap of apartment 5.3	VT3-1	Fiber	Blue	Unknown		0.93	6	6	0	0
	VT3-2	Fiber	Transparent	Cellulose		0.80				
	VT3-3	Fiber	Transparent	Polyester-Polyamide	0.39	0.95				
	VT3-4	Fiber	Transparent	Cellulose		0.82				
	VT3-5	Fragment	Transparent	Unknown		0.70				
	VT3-6	Fiber	Transparent	Cellulose		0.81				
	VT3-7	Fiber	Purple	Cellulose		0.86				
	VT3-8	Fiber	Transparent	Cellulose		0.80				

	VT3-9	Fiber	Transparent	Cellulose		0.90				
	VT3-10	Fiber	Red	Cellulose		0.74				
	VT3-11	Fiber	Transparent	Cellulose		0.86				
	VT3-12	Fiber	Transparent	Polyester	0.65	0.98				
	VT3-13	Fiber	Blue	Cellulose		0.74				
	VT3-14	Fiber	Transparent	Cellulose		0.84				
	VT3-15	Fiber	Transparent	Cellulose		0.86				
	VT3-16	Fiber	Transparent	Polyester	0.62	0.98				
	VT3-17	Fiber	Transparent	Cellulose		0.92				
	VT3-18	Fiber	Transparent	Polyester	0.46	0.94				
	VT3-19	Fiber	Transparent	Cellulose		0.81				
	VT3-20	Fiber	Transparent	Polypropylene	0.43	0.94				
	VT3-21	Fiber	Blue	Cellulose		0.74				
	VT3-22	Fiber	Transparent	Cellulose		0.80				
	VT3-23	Fiber	Transparent	Cellulose		0.81				
	VT3-24	Fiber	Transparent	Polyester	0.40	0.95				

	VT3-25	Fiber	Transparent	Cellulose		0.78				
	VT3-26	Fiber	Blue	Cellulose		0.92				

APARTMENT N. 6

Filter	Code	Shape	Color	Composition	Size (mm)	Score	TOT Plastics	MP	LMP	MAP
Tap of apartment 6.1	VO1-1	Fragment	Black	Arabic gum		0.64	1	1	0	0
	VO1-2	Fiber	Green	Ca carbonate		0.70				
	VO1-3	Fragment	Transparent	Unknown		0.72				
	VO1-4	Fragment	Transparent	Polyurethane	0.13	0.95				
	VO1-5	Fragment	Transparent	Cellulose		0.77				
	VO1-6	Fiber	Black	Cellulose		0.88				
	VO1-7	Fragment	Black	Aluminium sulphate		0.71				
	VO1-8	Fiber	Transparent	Cellulose		0.84				
	VO1-9	Fragment	White	Ca stearate		0.84				
Tap of apartment 6.2	VO2-1	Fiber	Transparent	Cellulose		0.87	1	1	0	0
	VO2-2	Fragment	Transparent	Talcum		0.77				

	VO2-3	Fiber	Transparent	Cellulose		0.75				
	VO2-4	Fiber	Blue	Cellulose		0.93				
	VO2-5	Fiber	Transparent	Cellulose		0.93				
	VO2-6	Fragment	Pink	Talcum		0.81				
	VO2-7	Fiber	Transparent	Cellulose		0.81				
	VO2-8	Fragment	Transparent	Cellulose		0.73				
	VO2-9	Fiber	Transparent	Cellulose		0.79				
	VO2-11	Fiber	Transparent	Cellulose		0.90				
	VO2-12	Fragment	Transparent	Unknown		0.87				
	VO2-13	Fiber	Transparent	Cellulose		0.79				
	VO2-14	Fragment	Transparent	Unknown		0.92				
	VO2-15	Fiber	Transparent	Cellulose		0.88				
	VO2-16	Fiber	Transparent	Cellulose		0.77				
	VO2-17	Fiber	Transparent	Unknown		0.86				
Tap of apartment 6.3	VO3-1	Fiber	Transparent	Cellulose		0.79	3	3	0	0
	VO3-2	Fiber	Transparent	Cellulose		0.88				

	VO3-3	Fiber	Transparent	Polyurethane	0.17	0.95				
	VO3-4	Fiber	Blue	Ca carbonate		0.84				
	VO3-5	Fiber	Transparent	Cellulose		0.78				
	VO3-6	Fiber	Black	Cellulose		0.90				
	VO3-7	Fragment	Transparent	Polyisoprene		0.83				
	VO3-8	Fiber	Transparent	Cellulose		0.77				
	VO3-9	Fiber	Blue	Polyester	0.38	0.98				
	VO3-10	Fiber	Blue	Polyamide	0.30	0.90				
	VO3-11	Fiber	Blue	Cellulose		0.84				
	VO3-12	Fiber	Transparent	Cellulose		0.83				
	VO3-13	Fiber	Transparent	Cellulose		0.86				
	VO3-14	Fiber	Transparent	Cellulose		0.82				
	VO3-15	Fiber	Blue	Cellulose		0.89				
	VO3-16	Fiber	Transparent	Aluminium silicate		0.72				
	VO3-17	Fiber	Black	Cellulose		0.91				
	VO3-18	Fragment	Transparent	Cellulose		0.86				

	VO3-19	Fiber	Transparent	Cellulose		0.82				
	VO3-20	Fiber	Transparent	Cellulose		0.74				
	VO3-21	Fragment	Transparent	Cellulose		0.70				
	VO3-22	Fiber	Transparent	Cellulose		0.91				
	VO3-23	Fiber	Transparent	Cellulose		0.92				
	VO3-24	Fiber	Pink	Cellulose		0.79				
	VO3-25	Fiber	Transparent	Cellulose		0.83				
	VO3-26	Fiber	Transparent	Cellulose		0.72				
	VO3-27	Fiber	Transparent	Cellulose		0.70				
	VO3-28	Fiber	Transparent	Cellulose		0.87				
	VO3-29	Fiber	Transparent	Cellulose		0.92				
	VO3-30	Fiber	Transparent	Cellulose		0.87				
	VO3-31	Fiber	Transparent	Cellulose		0.86				
	VO3-32	Fiber	Transparent	Cellulose		0.85				
	VO3-33	Fiber	Black	Cellulose		0.84				
	VO3-34	Fiber	Blue	Cellulose		0.92				

	VO3-35	Fiber	Blue	Cellulose		0.90				
	VO3-36	Fiber	Blue	Cellulose		0.78				
	VO3-37	Fragment	Pink	Unknown		0.90				

APARTMENT N. 7

Filter	Code	Shape	Color	Composition	Size (mm)	Score	TOT Plastics	MP	LMP	MAP
Tap of apartment 7.1	VSA1-1	Fragment	Transparent	Mg stearate		0.78	2	2	0	0
	VSA1-2	Fragment	Transparent	Unknown		0.93				
	VSA1-3	Fiber	Transparent	Cellulose		0.71				
	VSA1-4	Fiber	Transparent	Cellulose		0.83				
	VSA1-5	Fiber	Transparent	Cellulose		0.81				
	VSA1-6	Fiber	Transparent	Cellulose		0.81				
	VSA1-7	Fragment	Transparent	Ca stearate		0.70				
	VSA1-8	Fragment	Transparent	Ca stearate		0.72				
	VSA1-9	Fiber	Transparent	Cellulose		0.85				
	VSA1-10	Fiber	Transparent	Cellulose		0.71				

	VSA1-11	Fiber	Blue	Cellulose		0.90				
	VSA1-12	Fiber	Transparent	Unknown		0.86				
	VSA1-13	Fiber	Transparent	Cellulose		0.84				
	VSA1-14	Fragment	Transparent	Ca stearate		0.74				
	VSA1-15	Fiber	Transparent	Polyester	0.97	0.95				
	VSA1-16	Fiber	Transparent	Polyester	0.16	0.97				
	VSA1-17	Fiber	Transparent	Cellulose		0.83				
	VSA1-18	Fiber	Blue	Cellulose		0.70				
	VSA1-19	Fiber	Blue	Cellulose		0.76				
Tap of apartment 7.2	VSA2-1	Fiber	Black	Cellulose		0.79	1	1	0	0
	VSA2-2	Fiber	Transparent	Cellulose		0.75				
	VSA2-3	Fragment	Blue	Polyacrylate	0.09	0.78				
	VSA2-4	Fragment	Black	Cellulose		0.95				
	VSA2-5	Fiber	Transparent	Cellulose		0.88				
	VSA2-6	Fiber	Transparent	Cellulose		0.77				
	VSA2-7	Fiber	Black	Cellulose		0.82				

	VSA2-8	Fiber	Blue	Cellulose		0.77				
	VSA2-9	Fragment	Orange	Na and Al silicate		0.97				
	VSA2-10	Fiber	Transparent	Cellulose		0.92				
Tap of apartment 7.3	VSA3-1	Fiber	Transparent	Cellulose		0.81	3	3	0	0
	VSA3-2	Fiber	Transparent	Cellulose		0.80				
	VSA3-3	Fragment	Blue	Cellulose		0.75				
	VSA3-4	Fiber	Transparent	Cellulose		0.81				
	VSA3-5	Fragment	Transparent	Unknown		0.90				
	VSA3-6	Fragment	Grey	Aluminium sulphate		0.69				
	VSA3-7	Fiber	Blue	Cellulose		0.80				
	VSA3-8	Fragment	Grey	Acrylonitrile-butadiene-styrene	0.11	0.70				
	VSA3-9	Fiber	Transparent	Cellulose		0.72				
	VSA3-10	Fiber	Blue	Cellulose		0.71				
	VSA3-11	Fiber	Blue	Polyacrylate	0.17	0.70				
	VSA3-12	Fiber	Transparent	Cellulose		0.76				

	VSA3-13	Fiber	Black	Cellulose		0.90				
	VSA3-14	Fiber	Transparent	Polyester	0.99	0.97				
	VSA3-15	Fiber	Transparent	Unknown		0.83				
	VSA3-16	Fiber	Transparent	Cellulose		0.70				
	VSA3-17	Fiber	Transparent	Cellulose		0.73				
	VSA3-18	Fragment	Grey	Aluminium sulphate		0.70				
	VSA3-19	Fiber	Transparent	Cellulose		0.78				
	VSA3-20	Fiber	Transparent	Cellulose		0.90				
	VSA3-21	Fiber	Transparent	Cellulose		0.87				

APARTMENT N. 8

Filter	Code	Shape	Color	Composition	Size (mm)	Score	TOT Plastics	MP	LMP	MAP
Tap of apartment 8.1	VS1-1	Fiber	Transparent	Cellulose		0.91	2	2	0	0
	VS1-2	Fiber	Transparent	Cellulose		0.85				
	VS1-3	Fiber	Transparent	Polyester	0.41	0.96				
	VS1-4	Fiber	Black	Cellulose		0.77				

	VS1-5	Fiber	Black	Cellulose		0.78				
	VS1-6	Fiber	Transparent	Cellulose		0.75				
	VS1-7	Fiber	Black	Cellulose		0.84				
	VS1-8	Fiber	Blue	Cellulose		0.88				
	VS1-9	Fiber	Transparent	Cellulose		0.79				
	VS1-10	Fiber	Transparent	Cellulose		0.81				
	VS1-11	Fiber	Blue	Unknown		0.89				
	VS1-12	Fiber	Black	Cellulose		0.80				
	VS1-13	Fiber	Blue	Aluminium sulphate		0.69				
	VS1-14	Fiber	Transparent	Cellulose		0.80				
	VS1-15	Fiber	Transparent	Cellulose		0.80				
	VS1-16	Fiber	Transparent	Cellulose		0.93				
	VS1-17	Fiber	Transparent	Cellulose		0.78				
	VS1-18	Fiber	Blue	Cellulose		0.77				
	VS1-19	Fiber	Black	Cellulose		0.85				
	VS1-20	Fiber	Red	Cellulose		0.72				

	VS1-21	Fiber	Transparent	Polyamide	0.57	0.96				
	VS1-22	Fiber	Transparent	Cellulose		0.91				
	VS1-23	Fiber	Blue	Unknown		0.87				
	VS1-24	Fiber	Transparent	Cellulose		0.87				
	VS1-25	Fiber	Transparent	Cellulose		0.78				
	VS1-26	Fiber	Blue	Cellulose		0.64				
	VS1-27	Fiber	Transparent	Cellulose		0.85				
	VS1-28	Fiber	Black	Cellulose		0.79				
	VS1-29	Fiber	Transparent	Cellulose		0.91				
	VS1-30	Fiber	Transparent	Cellulose		0.95				
	VS1-31	Fiber	Transparent	Cellulose		0.80				
	VS1-32	Fiber	Transparent	Cellulose		0.75				
	VS1-33	Fiber	Transparent	Cellulose		0.88				
	VS1-34	Fiber	Transparent	Cellulose		0.87				
	VS1-35	Fiber	Transparent	Cellulose		0.91				
	VS1-36	Fiber	Transparent	Cellulose		0.82				

	VS1-37	Fiber	Blue	Cellulose		0.82				
	VS1-38	Fiber	Transparent	Cellulose		0.72				
	VS1-39	Fiber	Transparent	Cellulose		0.71				
	VS1-40	Fiber	Transparent	Cellulose		0.76				
	VS1-41	Fiber	Transparent	Cellulose		0.77				
	VS1-42	Fiber	Transparent	Cellulose		0.81				
	VS1-43	Fiber	Black	Cellulose		0.94				
	VS1-44	Fiber	Blue	Cellulose		0.89				
	VS1-45	Fragment	Transparent	Cellulose		0.73				
	VS1-46	Fiber	Transparent	Cellulose		0.88				
	VS1-47	Fiber	Transparent	Cellulose		0.81				
	VS1-48	Fiber	Transparent	Cellulose		0.72				
	VS1-49	Fiber	Blue	Cellulose		0.88				
	VS1-50	Fiber	Blue	Cellulose		0.91				
	VS1-51	Fiber	Blue	Cellulose		0.87				
	VS1-52	Fiber	Transparent	Cellulose		0.70				

	VS1-53	Fiber	Transparent	Cellulose		0.87				
	VS1-54	Fiber	Transparent	Cellulose		0.71				
	VS1-55	Fiber	Transparent	Cellulose		0.79				
	VS1-56	Fragment	Black	Ca carbonate		0.78				
	VS1-57	Fiber	Transparent	Cellulose		0.93				
	VS1-58	Fiber	Transparent	Cellulose		0.76				
	VS1-59	Fiber	Transparent	Cellulose		0.90				
	VS1-60	Fiber	Transparent	Cellulose		0.78				
	VS1-61	Fiber	Transparent	Unknown		0.94				
	VS1-62	Fiber	Transparent	Cellulose		0.75				
	VS1-63	Fiber	Transparent	Cellulose		0.88				
	VS1-64	Fragment	Transparent	Cellulose		0.87				
	VS1-65	Fiber	Transparent	Cellulose		0.82				
	VS1-66	Fragment	Transparent	Cellulose		0.88				
	VS1-67	Fiber	Transparent	Cellulose		0.63				
	VS1-68	Fiber	Transparent	Cellulose		0.85				

	VS1-69	Fiber	Blue	Cellulose		0.75				
	VS1-70	Fiber	Transparent	Cellulose		0.88				
	VS1-71	Fiber	Transparent	Cellulose		0.77				
	VS1-72	Fiber	Transparent	Cellulose		0.86				
	VS1-73	Fiber	Transparent	Cellulose		0.82				
	VS1-74	Fiber	Transparent	Cellulose		0.84				
	VS1-75	Fragment	Transparent	Unknown		0.82				
	VS1-76	Fiber	Transparent	Cellulose		0.86				
	VS1-77	Fiber	Transparent	Cellulose		0.90				
	VS1-78	Fiber	Transparent	Cellulose		0.82				
	VS1-79	Fiber	Black	Cellulose		0.85				
	VS1-80	Fiber	Transparent	Cellulose		0.85				
Tap of apartment 8.2	VS2-1	Fiber	Transparent	Cellulose		0.82	2	2	0	0
	VS2-2	Fiber	Transparent	Ca carbonate		0.77				
	VS2-3	Fiber	Transparent	Cellulose		0.87				
	VS2-4	Fiber	Transparent	Cellulose		0.85				

	VS2-5	Fiber	Blue	Polyester	0.50	0.78				
	VS2-6	Fiber	Transparent	Cellulose		0.82				
	VS2-7	Fiber	Transparent	Cellulose		0.76				
	VS2-8	Fiber	Transparent	Cellulose		0.80				
	VS2-9	Fiber	Transparent	Cellulose		0.81				
	VS2-10	Fiber	Transparent	Polyester	0.59	0.89				
	VS2-11	Fiber	Blue	Cellulose		0.82				
	VS2-12	Pellet	Transparent	Ca carbonate		0.99				
	VS2-13	Fiber	Black	Unknown		0.82				
	VS2-14	Fiber	Red	Cellulose		0.73				
	VS2-15	Fiber	Transparent	Cellulose		0.82				
	VS2-16	Fragment	Transparent	Unknown		0.94				
	VS2-17	Fiber	Transparent	Cellulose		0.91				
Tap of apartment 8.3	VS3-1	Fiber	Blue	Cellulose		0.76	2	2	0	0
	VS3-2	Fiber	Transparent	Cellulose		0.85				
	VS3-3	Fiber	Black	Cellulose		0.91				

	VS3-4	Fiber	Transparent	Cellulose		0.79				
	VS3-5	Fiber	Transparent	Cellulose		0.95				
	VS3-6	Fiber	Black	Cellulose		0.90				
	VS3-7	Fiber	Blue	Cellulose		0.82				
	VS3-8	Fiber	Black	Cellulose		0.73				
	VS3-9	Fiber	Transparent	Cellulose		0.91				
	VS3-10	Fiber	Blue	Cellulose		0.81				
	VS3-11	Fiber	Black	Cellulose		0.72				
	VS3-13	Fragment	Blue	Polyacrylate	0.20	0.71				
	VS3-14	Fiber	Black	Cellulose		0.74				
	VS3-15	Fiber	Transparent	Cellulose		0.89				
	VS3-16	Fragment	Transparent	Polypropylene	0.20	0.98				
	VS3-17	Fiber	Blue	Cellulose		0.70				

APARTMENT N. 9

Filter	Code	Shape	Color	Composition	Size (mm)	Score	TOT Plastics	MP	LMP	MAP
Tap of apartment 9.1	VVE1-1	Fiber	Black	Cellulose		0.84	0	0	0	0
	VVE1-2	Fiber	Transparent	Cellulose		0.71				
	VVE1-3	Fiber	Red	Cellulose		0.78				
	VVE1-4	Fiber	Transparent	Cellulose		0.87				
	VVE1-5	Fiber	Transparent	Cellulose		0.86				
	VVE1-6	Fiber	Transparent	Cellulose		0.88				
	VVE1-7	Fiber	Transparent	Cellulose		0.77				
	VVE1-8	Fiber	Transparent	Cellulose		0.91				
	VVE1-9	Fiber	Blue	Cellulose		0.85				
	VVE1-10	Fragment	Transparent	Ca stearate		0.79				
	VVE1-11	Fiber	Transparent	Cellulose		0.86				
	VVE1-12	Fiber	Transparent	Cellulose		0.93				
	VVE1-13	Fragment	Transparent	Ca stearate		0.77				
	VVE1-14	Fiber	Transparent	Cellulose		0.73				

	VVE1-15	Fiber	Purple	Cellulose		0.84				
	VVE1-16	Fiber	Transparent	Cellulose		0.82				
	VVE1-17	Fiber	Transparent	Cellulose		0.76				
	VVE1-18	Fiber	Black	Cellulose		0.87				
	VVE1-19	Fiber	Transparent	Polyisoprene		0.82				
Tap of apartment 9.2	VVE2-1	Fiber	Transparent	Cellulose		0.78	2	2	0	0
	VVE2-2	Fiber	Transparent	Cellulose		0.81				
	VVE2-3	Fragment	Blue	Polyacrylate	0.07	0.74				
	VVE2-4	Fragment	Grey	Quartz		0.67				
	VVE2-5	Fragment	Grey	Quartz		0.90				
	VVE2-6	Fiber	Transparent	Cellulose		0.90				
	VVE2-7	Fragment	Grey	Quartz		0.91				
	VVE2-8	Fiber	Blue	Cellulose		0.87				
	VVE2-9	Fiber	Transparent	Cellulose		0.80				
	VVE2-10	Fragment	Grey	Quartz		0.75				
	VVE2-11	Fiber	Black	Polyamide	0.38	0.93				

	VVE2-12	Fiber	Transparent	Cellulose		0.75				
	VVE2-13	Fiber	Transparent	Cellulose		0.77				
	VVE2-14	Fragment	Red	Ca carbonate		0.80				
	VVE2-15	Fiber	Transparent	Cellulose		0.80				
	VVE2-16	Fiber	Black	Cellulose		0.81				
	VVE2-17	Fiber	Transparent	Cellulose		0.84				
	VVE2-18	Fiber	Black	Cellulose		0.88				
	VVE2-19	Fiber	Transparent	Cellulose		0.71				
	VVE2-20	Fiber	Transparent	Cellulose		0.84				
	VVE2-21	Fiber	Transparent	Cellulose		0.78				
	VVE2-22	Fiber	Red	Cellulose		0.72				
	VVE2-23	Fiber	Transparent	Cellulose		0.92				
	VVE2-24	Fiber	Transparent	Cellulose		0.74				
	VVE2-25	Fiber	Transparent	Cellulose		0.86				
	VVE2-26	Fiber	Blue	Cellulose		0.71				
Tap of apartment 9.3	VVE3-1	Fiber	Transparent	Cellulose		0.89	2	1	1	0

	VVE3-2	Fiber	Transparent	Cellulose		0.93				
	VVE3-3	Fragment	Purple	Cellulose		0.90				
	VVE3-4	Fiber	Transparent	Cellulose		0.83				
	VVE3-5	Fragment	Transparent	Unknown		0.91				
	VVE3-6	Fiber	Transparent	Cellulose		0.74				
	VVE3-7	Fiber	Blue	Cellulose		0.70				
	VVE3-8	Fiber	Transparent	Cellulose		0.79				
	VVE3-9	Fiber	Transparent	Cellulose		0.88				
	VVE3-10	Fiber	Transparent	Cellulose		0.79				
	VVE3-11	Fiber	Transparent	Cellulose		0.74				
	VVE3-12	Fragment	Grey	Quartz		0.76				
	VVE3-13	Fragment	Grey	Quartz		0.70				
	VVE3-14	Fiber	Transparent	Cellulose		0.70				
	VVE3-15	Fiber	Transparent	Polyester	0.30	0.93				
	VVE3-16	Fiber	Transparent	Cellulose		0.87				
	VVE3-17	Fiber	Transparent	Cellulose		0.70				

	VVE3-18	Fragment	Grey	Aluminium sulphate		0.69				
	VVE3-19	Fragment	Grey	Aluminium sulphate		0.70				
	VVE3-20	Fiber	Transparent	Polyester	4.05	0.93				
	VVE3-21	Fiber	Red	Cellulose		0.77				
	VVE3-22	Fiber	Red	Cellulose		0.85				

APARTMENT N. 10

Filter	Code	Shape	Color	Composition	Size (mm)	Score	TOT Plastics	MP	LMP	MAP
Tap of apartment 10.1	VB1-1	Fiber	Transparent	Cellulose		0.81	2	2	0	0
	VB1-2	Fiber	Blue	Cellulose		0.76				
	VB1-3	Fiber	Blue	Cellulose		0.84				
	VB1-4	Fiber	Transparent	Cellulose		0.77				
	VB1-5	Fiber	Transparent	Cellulose		0.77				
	VB1-6	Fiber	Transparent	Cellulose		0.87				
	VB1-7	Fiber	Transparent	Cellulose		0.77				
	VB1-9	Fragment	White	Ca carbonate		0.88				

	VB1-10	Fiber	Transparent	Cellulose		0.76				
	VB1-11	Fiber	Transparent	Cellulose		0.79				
	VB1-12	Fiber	Transparent	Cellulose		0.73				
	VB1-13	Fiber	Transparent	Cellulose		0.84				
	VB1-14	Fiber	Transparent	Cellulose		0.79				
	VB1-15	Fiber	Transparent	Cellulose		0.88				
	VB1-16	Fiber	Transparent	Cellulose		0.75				
	VB1-17	Fiber	Transparent	Cellulose		0.71				
	VB1-18	Fiber	Transparent	Cellulose		0.78				
	VB1-19	Fiber	Blue	Cellulose		0.69				
	VB1-20	Fiber	Black	Cellulose		0.82				
	VB1-21	Fiber	Black	Unknown		0.92				
	VB1-22	Fiber	Transparent	Cellulose		0.93				
Tap of apartment 10.2	VB2-1	Fiber	Transparent	Cellulose		0.87	1	1	0	0
	VB2-2	Fiber	Black	Cellulose		0.82				
	VB2-3	Fiber	Transparent	Cellulose		0.72				

	VB2-4	Fiber	Transparent	Cellulose		0.77				
	VB2-5	Fiber	Transparent	Cellulose		0.76				
	VB2-6	Fiber	Transparent	Cellulose		0.72				
	VB2-7	Fiber	Transparent	Cellulose		0.87				
	VB2-8	Fiber	Transparent	Polyester	0.36	0.98				
	VB2-9	Fiber	Transparent	Cellulose		0.80				
	VB2-10	Fiber	Transparent	Cellulose		0.87				
	VB2-11	Fiber	Blue	Cellulose		0.75				
	VB2-12	Fragment	Transparent	Cellulose		0.73				
	VB2-13	Fiber	Transparent	Cellulose		0.73				
	VB2-14	Fiber	Transparent	Cellulose		0.84				
	VB2-15	Fiber	Black	Cellulose		0.89				
	VB2-16	Fiber	Transparent	Cellulose		0.82				
Tap of apartment 10.3	VB3-1	Fiber	Transparent	Cellulose		0.82	4	4	0	0
	VB3-2	Fiber	Transparent	Cellulose		0.76				
	VB3-3	Fiber	Transparent	Cellulose		0.77				

	VB3-4	Fiber	Transparent	Cellulose		0.92				
	VB3-5	Fiber	Blue	Polyamide	0.26	0.89				
	VB3-6	Fiber	Transparent	Cellulose		0.81				
	VB3-7	Fiber	Transparent	Cellulose		0.78				
	VB3-8	Fiber	Transparent	Cellulose		0.76				
	VB3-9	Fiber	Transparent	Cellulose		0.87				
	VB3-10	Fragment	Transparent	Unknown		0.90				
	VB3-11	Fiber	Transparent	Cellulose		0.87				
	VB3-12	Fiber	Transparent	Cellulose		0.78				
	VB3-15	Fiber	Transparent	Cellulose		0.84				
	VB3-16	Fiber	Transparent	Cellulose		0.72				
	VB3-17	Fiber	Transparent	Polyester	0.96	0.93				
